# Adropin correlates with aging-related neuropathology in humans and improves cognitive function in aging mice

**DOI:** 10.1038/s41514-021-00076-5

**Published:** 2021-08-30

**Authors:** Subhashis Banerjee, Sarbani Ghoshal, Clemence Girardet, Kelly M. DeMars, Changjun Yang, Michael L. Niehoff, Andrew D. Nguyen, Prerana Jayanth, Brittany A. Hoelscher, Fenglian Xu, William A. Banks, Kim M. Hansen, Jinsong Zhang, Eduardo Candelario-Jalil, Susan A. Farr, Andrew A. Butler

**Affiliations:** 1 Department of Pharmacology and Physiology, Saint Louis University School of Medicine, St. Louis, MO USA; 2 Department of Neuroscience, McKnight Brain Institute, University of Florida, Gainesville, FL USA; 3 Division of Geriatric Medicine, Saint Louis University School of Medicine, St. Louis, MO USA; 4 Henry and Amelia Nasrallah Center for Neuroscience, Saint Louis University School of Medicine, St. Louis, MO USA; 5 Department of Biology, College of Arts and Sciences, Saint Louis University, St. Louis, MO USA; 6 Geriatrics Research Education and Clinical Center, Veterans Affairs Puget Sound Health Care System, Seattle, WA USA; 7 Division of Gerontology and Geriatric Medicine, Department of Medicine, University of Washington School of Medicine, Seattle, WA USA; 8 Saint Louis Veterans Affairs Medical Center, Research Service, John Cochran Division, St. Louis, MO USA; 9 Present Address: Department of Biological Sc. and Geology, QCC-CUNY, Bayside, NY USA; 10 Present Address: Sorbonne Université, Paris, France

**Keywords:** Cognitive ageing, Risk factors

## Abstract

The neural functions of adropin, a secreted peptide highly expressed in the brain, have not been investigated. In humans, adropin is highly expressed in astrocytes and peaks during critical postnatal periods of brain development. Gene enrichment analysis of transcripts correlating with adropin expression suggests processes relevant to aging-related neurodegenerative diseases that vary with age and dementia state, possibly indicating survivor bias. In people aged <40 y and ‘old-old’ (>75 y) diagnosed with dementia, adropin correlates positively with genes involved in mitochondrial processes. In the ‘old-old’ without dementia adropin expression correlates positively with morphogenesis and synapse function. Potent neurotrophic responses in primary cultured neurons are consistent with adropin supporting the development and function of neural networks. Adropin expression in the ‘old-old’ also correlates positively with protein markers of tau-related neuropathologies and inflammation, particularly in those without dementia. How variation in brain adropin expression affects neurological aging was investigated using old (18-month) C57BL/6J mice. In mice adropin is expressed in neurons, oligodendrocyte progenitor cells, oligodendrocytes, and microglia and shows correlative relationships with groups of genes involved in neurodegeneration and cellular metabolism. Increasing adropin expression using transgenesis improved spatial learning and memory, novel object recognition, resilience to exposure to new environments, and reduced mRNA markers of inflammation in old mice. Treatment with synthetic adropin peptide also reversed age-related declines in cognitive functions and affected expression of genes involved in morphogenesis and cellular metabolism. Collectively, these results establish a link between adropin expression and neural energy metabolism and indicate a potential therapy against neurological aging.

## Introduction

Aging associates with declining cognitive performance and capacity for tracking the “what/where/when” in our daily activities (episodic memory)^1–3^. Mild Cognitive Impairment (MCI) describes a condition in which impairments in organizing and remembering daily activities are perceptible to individuals but are not severe enough to cause loss of independence^4,5^. Increased risk for dementia and observations of dementia-related changes in the brain suggests MCI is an early stage in the spectrum of life-threatening dementias, of which Late-Onset Alzheimer’s Disease (LOAD) is the most common^6–9^. Demographic changes resulting in an aging population this century will increase the prevalence of MCI and LOAD^10^. Identifying new treatments targeting aging-related changes in brain structure that underly dementia is thus considered an important goal.

Short open reading frames encoding small polypeptides are a potentially rich source of new drug targets^11,12^. Adropin^1-^^76^ is encoded by the Energy Homeostasis Associated (*ENHO*) gene, and was identified by Genentech’s Secreted Protein Discovery Initiative^13^. Modelling in silico suggests a signal sequence (adropin^1-33^) targets the secretory pathway^14–16^. The predicted secreted domain (adropin^34-^^76^) is sufficient for biological activity in cultured cells and rodent models^14,17–26^. Reports of adropin immunoreactivity in the circulation of humans, nonhuman primates (NHP) and rodents are consistent with a secreted peptide with endocrine functions^14,27,28^. However, the exact sequence and any posttranslational modifications of the mature peptide are not known.

Experiments using mouse models suggest adropin regulates metabolic processes in the periphery^14,19,26,29,30^. However, several observations suggest adropin is a neuropeptide. Expression of the *ENHO* transcript is orders of magnitude higher in the brain relative to non-neural tissues^14,28,31–34^. The two candidates for cell-surface adropin receptors are also highly expressed in the CNS, and each appears to or is known to regulate neural development. The orphan G protein coupled receptor GPR19 is obligatory for adropin activity^21,24,25^ and is also highly expressed in the CNS relative to other tissues^35,36^. High GPR19 expression in the embryonic brain suggests a role in development^37^. However, evidence for coupling of adropin with GPR19 is controversial^38^. Adropin also interacts with NB-3/Contactin6 (CNTN6) in protein-protein yeast two hybrid assays^31^. CNTN6 is a brain-specific non-canonical membrane-tethered Notch1 ligand that belongs to the immunoglobulin cell adhesion molecule superfamily (IgCAMs). Contactins function as cell-adhesion proteins in the developing nervous system and regulate axonal guidance, development of neurites, synapse formation, synaptic plasticity, and neural regeneration^39^.

Recent studies suggest adropin may regulate processes in the nervous system that could affect aging. Aging-related declines of plasma adropin concentrations and protein expression in the brain of Sprague Dawley rats correlate with the expression of protein markers of oxidative stress^40^. Plasma concentrations in humans also decline with aging, particularly in males^41^. In NHP tissues, expression of the adropin transcript correlates with genes associated with aging-related neurodegeneration^28^. Here, we investigate the relationships between adropin expression and aging-related cognitive decline. Results from the analysis of open access transcriptome from humans and intervention experiments using mouse models indicate that adropin is a potential lead for developing new treatments againsts cognitive decline commonly observed with advanced aging.

## Results

### ENHO expression in human tissue samples

**Fig. 1:**
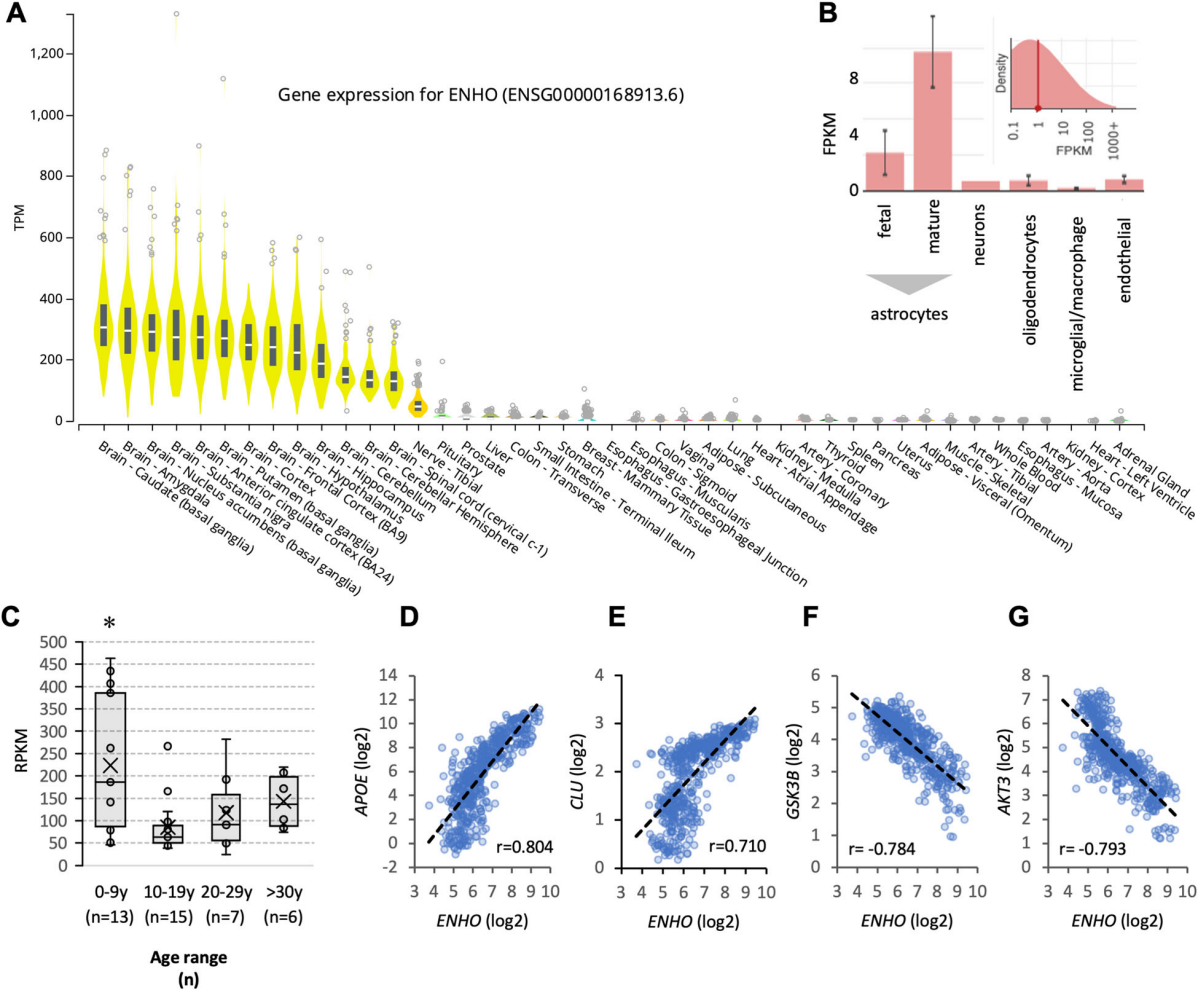
*ENHO* expression in the human brain. **A***ENHO* transcript expression (TPM) in human tissues ranked high to low reading left to right (GTEx). **B** *ENHO* expression in isolated cell populations (brain.org). **C** *ENHO* expression by age group. This analysis used data from 41 individuals (mean ± SD for age, 15.8 ± 11.2 y, range 4 mo–40 y; 22 males, 14.3 ± 11.7 y, 4 mo–37 y; 19 females, 17.5 ± 10.6 y, 1–40 y) downloaded from The Atlas of the Developing Human Brain^28,30^. Multiple samples from different brain regions were obtained from individuals; mean expression for samples from individuals was used for the analysis. Data were pooled into 10-year bins (birth-9 y, 10–19 y, 20–29 y, and >30 y). *p < 0.05 vs. all other groups combined. **D**–**E** Expression of *APOE* (**D**), Clusterin/ApoJ (**E**), *GCK3B* (**F**), and *AKT3* (**G**) related to ENHO (524 samples from 41 individuals, data from The Atlas of the Developing Human Brain^28,30^).

We first compared *ENHO* expression between human tissues using The Atlas of the Developing Human Brain (www.brainspan.org) and Genotype-Tissue Expression (GTEx) projects. *ENHO* is highly expressed in all brain structures relative to other tissues (Fig. 1A) and is moderately (10%) higher in some areas of the male brain when compared to females (Fig. S1, S2A). *ENHO* expression is highest in mature astrocytes, with lower expression in fetal astrocytes, neurons, oligodendrocytes, and endothelial cells (Fig. 1B)^42^. *ENHO* expression appears to peak in the first decade of life (Fig. 1C) and is then constant until the 8th decade of life (Fig. 1C and in GTEx data not shown).

### ENHO expression correlates with energy metabolism and LOAD risk genes in the human brain

**Fig. 2:**
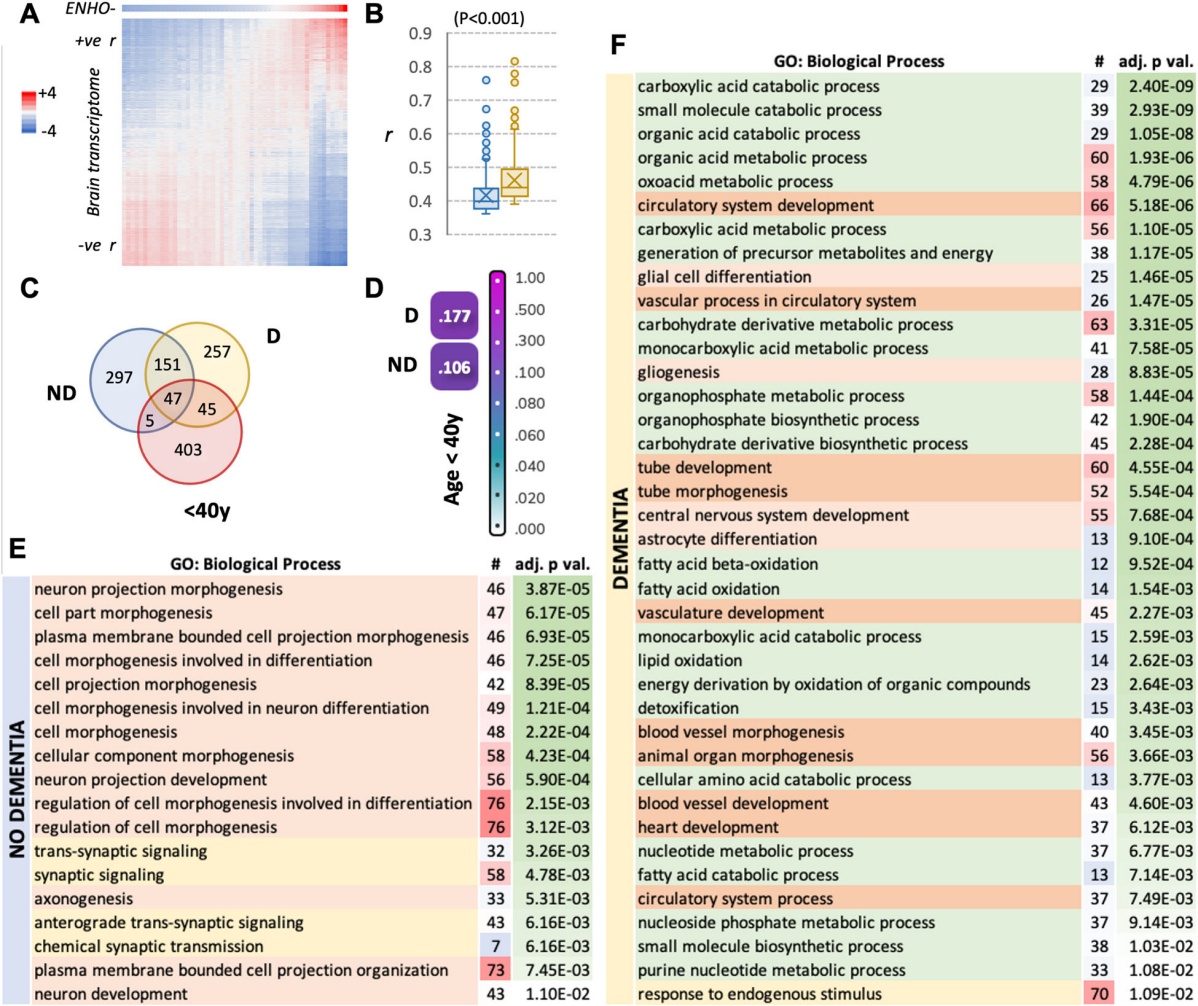
Correlations between ENHO and gene networks differs between dementia state of the old-old. **A** Correlation matrix heat map showing the relationships between *z*-scores for *ENHO* expression and the brain transcriptome. The matrix is representative and was derived from The Atlas of the Developing Human Brain. Genes with a mean expression level (RPKM) of >10 were selected; each pixel is a bin comprised of the averages of ten samples, with ten genes in each sample. **B** Correlation coefficients (r) between expression of *ENHO* and the top 1% of genes. ENHO expression shows a tighter correlation, indicated by higher r, in brain samples isolated from the old-old who diet with a dementia diagnosis (‘D’) compared to the old-old without a dementia diagnosis (‘ND’). **C** Venn diagram showing overlap in the top 500 genes correlating with ENHO expression (ranked by correlation coefficient) for the ND group, D group and people aged < 40 years (< 40 y). **D** Comparison of biological processes correlating with ENHO expression in the ND group, D group and people aged < 40 years. A score > 0.1 indicates a significant correlation between groups; the D and < 40 y groups appear to be more closely related. **E**, **F** GO:Biological Processes correlating with ENHO expression in ND group (**D**) or D groups (**E**). ‘#’refers to the number of hits (genes); processes with an adjusted *p* value of < 0.01 are shown. Biological processes are color coded (orange for neural development, green for metabolism, yellow for signaling).

A correlation matrix was used to identify gene networks co-regulated with the *ENHO* transcript using data downloaded from The Atlas of the Developing Human Brain^28,30^ (Fig. 2A). These data are from 41 individuals (mean ± SD age, 15.8 ± 11.2 y, range 4 mo–40 y); 22 were male (14.3 ± 11.7 y, 4 mo–37 y) and 19 were female (17.5 ± 10.6 y, 1–40 y). Samples from multiple brain structures were obtained for each individual. For the correlation analysis the entire dataset (524 samples) was used. Coefficients (r) comparing *ENHO* and all other genes expressed were ranked high to low by *r*. Using *r* > 0.7 as an arbitrary cut-off to define genes exhibiting similar expression profiles identified 838 genes meeting the selection criteria.

Experiments using mouse and organ explants suggests that adropin regulates cellular energy metabolism^17,18,26^. Gene enrichment analysis suggests ENHO expression correlates positively with processes related to reduction-oxidation (redox) reactions and mitochondrial activity. Top ranked GO:molecular functions included “oxidoreductase activity” (*q* value FDR, 2.11 × 10^−18^; 86 gene hits/783 genes in genome), “electron transfer activity” (2.37 × 10^−10^, 28/151), “oxidoreductase activity, acting on NAD(P)H, quinone or similar compound as acceptor” (8.08 × 10^−7^, 15/61), and “NAD(P)H dehydrogenase (quinone) activity” (2.69 × 10^−5^, 12/49). Top GO:biological processes included “small molecule catabolic process” (2.22 × 10^−11^, 52/452), and “generation of precursor metabolites and energy” (6.99 × 10^−11^, 58/571). Top ranked GO:cellular components were mitochondrial: “mitochondrion” (8.04 × 10^−23^, 153/1897) “mitochondrial inner membrane” (4.62 × 10^−18^, 68/556), “organelle inner membrane” (5.44 × 10^−17^, 70/617) and “mitochondrial envelope” (8.98 × 10^−17^, 83/842).

A candidate gene approach identified strong positive correlations between *ENHO* and *APOE* or clusterin (CLU) (Fig. 1D, E). APOE variants (e2, e3, e4) are the strongest genetic risk factors identified for LOAD, with the e4 allele increasing risk and e2 allele conferring protection^43,44^. Clusterin (CLU, also known as ApoJ) is an extracellular chaperone linked to Aβ clearance and toxicity^45^. APOE and CLU are highly expressed by astrocytes and, as predicted based on expression data showing high ENHO expression in astrocytes (Fig. 1B), the gene set correlating with ENHO was highly enriched for astrocytic and glial markers (for e.g., ToppCell Atlas “Visual_Cortex-Non-neuronal-Astrocyte|Visual_Cortex/Region, Lineage, Class, Subclass and Cluster” *q* value FDR, 2.65 × 10–98; 85 hits out of 195 identified genes).

Strong negative correlations were also observed between *ENHO* and either *GSK3B* (Fig. 1F) or *AKT3* (Fig. 1G), which is the predominant AKT signaling isoform upstream of GSK3B expressed in the brain^46^. Tau phosphorylation by glycogen synthase kinase 3 (GSK3) contributes to development of neurofibrillary lesions observed in LOAD^47^.

### ENHO relationships with other genes in the ‘old-old’ are specific for dementia status

Whether advanced aging and dementia affects *ENHO* expression was investigated using data from The Aging, Dementia and TBI Study (http://aging.brain-map.org/). This study involved 107 people selected from the Adult Changes in Thought (ACT) cohort^48^, a population-based, prospective analysis of neurological aging^49–52^. Selected participants were mostly male (63 males, 44 females), varied in years of education and dementia status and age (77–102 y at time of death, median 90 y); 52 were diagnosed with dementia (D) by the time of death (30 with AD; 12 with dementia of multiple etiologies; four with vascular dementia). Postmortem samples were collected from brain regions known to exhibit neurodegeneration and pathology resulting from LOAD and Lewy body disease: frontal white matter (FWM), hippocampus (HIP), parietal cortex, and temporal cortex (TCx).

We first compared *ENHO* expression between brain structures and between individuals based on dementia state and APOE allele. *ENHO* expression is not affected by dementia status in humans (Fig. S2B). While these data suggest neurodegenerative diseases do not associate with large differences in *ENHO* expression, a trend (*p* < 0.1) was noted for higher *ENHO* expression in TCx and FWM samples from participants with >1 APOEe4 allele (Fig. S2C). Correlations between brain structures in the expression of gene networks suggests regulation by systemic factors^48^. We observed correlations in *ENHO* expression between brain structures (Fig. S2D-K), suggesting that common transcriptional and/or post-transcriptional regulatory elements affect expression throughout the nervous system.

The correlation approach was used to identify gene networks including *ENHO* to groups divided by dementia diagnosis at the time of specimen collection (Dementia or No Dementia, hereafter referred to as D and ND). The rationale for separating data into two groups is based on relationships between neurodegenerative disease state, RNA quality and agonal conditions that can result from ischemia; these conditions can complicate gene expression data analysis in postmortem brain samples^48,53,54^.

Aging adversely affect histone structure and transcription factor function^55,56^. In the old-old group, very few genes met the previously used selection criteria (*r* > 0.7) (Fig. 2B), consistent with declining fidelity in gene regulation. We therefore used the top 1% of genes (500 out of ~50,200) correlating with the *ENHO* transcript. Surprisingly, correlation coefficients between *ENHO* and genes ranked in the top 1% are significantly higher in people with D compared to ND (Fig. 2B). Significant overlap is observed in genes correlating positively with *ENHO* in the D and ND groups, with 40% (198/500) genes within each population found in both clusters (Fig. 2C). Comparing the top 500 genes correlating with *ENHO* in the The Atlas of the Developing Human Brain (people aged < 40 y) suggests that a closer relationship exists with the D group relative ND group (Fig. 2C). Out of 500 genes, 92 (18%) were common to the <40 y and D groups while only 52 (10%) were common to the <40 y and ND groups. A comparison of the biological processes correlating with ENHO also indicated a closer relationship between the D and <40 y groups (Fig. 2D).

Using stringent selection criterion (adj. *p* < 0.05) for gene enrichment analysis indicated differences in biological processes correlating with *ENHO* in the D and ND groups. In the ND group, neural development dominated the list of biological processes (Fig. 2E). GO:Cellular Component for the D group also related to neurons such as “somatodendritic compartment”. In contrast, metabolic processes dominated in the D group (Fig. 2F). It was also interesting to note processes related to vascular function and development (for e.g., “circulatory system development”, “tube morphogenesis” and “blood vessel development”) are observed in the D group. This is relevant given data from animal and clinical studies showing a positive relationship between adropin expression and vascular function^40,57–59^. GO:Cellular Component for the D group appeared to be predominantly mitochondrial (“mitochondrion”, “mitochondrial membrane”) and cellular processes related to the endoplasmic reticulum such as “organelle development” and “Golgi apparatus”).

To avoid bias associated with selecting small groups of genes, an unbiased screen applied multiple enrichment tools to the entire dataset of genes. As before, data from the D and ND groups were separated for the analysis (http://pharmacology.slu.edu/results/andrew/TBI_Dementia/). Genome-wide gene set enrichment revealed biological pathways either enriched or depleted in a manner correlated with *ENHO* expression. Four independent studies were performed to include (i) all 5529 biological pathways, designated “All pathways”, or a subset of these pathways belonging to (ii) REACTOME, designated “REACTOME pathways”, (iii) KEGG, designated “KEGG pathways”, or (iv) the remaining pathways, designated “non-REACTOME-KEGG pathways”. Pathways positively correlated with *ENHO* expression were shown by positive NESs (normalized enrichment scores). Pathways negatively correlated with *ENHO* expression shown by negative NESs.

The results from this analysis also identified strong correlations between adropin expression and energy metabolism. Specifically, *ENHO* expression positively associates with catabolic processes such as “KEGG_OXIDATIVE_PHOSPHORYLATION”, “KEGG_FATTY_ACID_METABOLISM” and “REACTOME_RESPIRATORY_ELECTRON_TRANSPORT”. In contrast, negative associations included anabolic pathways such as “KEGG_STEROID_BIOSYNTHESIS”, “KEGG_RIBOSOME”, “KEGG_DNA_REPLICATION”, and “REACTOME_CHOLESTEROL_BIOSYNTHESIS”. Interestingly, high *ENHO* expression was also associated with low expression of inflammatory pathways such as “REACTOME_INFLAMMASOMES” and “REACTOME_THE_NLRP3_INFLAMMASOME”. *ENHO* expression in the human brain also correlated with common neurodegenerative disorders (Parkinson’s disease, Huntington’s disease, and Alzheimer’s disease). This was shown by the significant positive or negative enrichment of “KEGG_PARKINSONS_DISEASE”, “KEGG_HUNTINGTONS_DISEASE” and “KEGG_ALZHEIMERS_DISEASE”, or the related pathways in *ENHO*-expressing or ENHO-depleted patient samples.

The correlations between *ENHO* expression and neurotrophic processes suggests functions related to brain development, an observation consistent with deficits in neural development reported in adropin knockout mice^31^. *ENHO* expression in ‘old-old’ humans appears to associate with increased expression of genes involved in mitochondrial and neurotrophic processes, suggesting that higher expression may benefit patients with advanced age. Indeed, enrichment of neurodegenerative pathways in genes correlating with ENHO expression appears stronger in D patients compared to ND patients. Enrichment of these pathways also appears stronger in female compared to male patients, suggesting sex differences in the robustness of gene networks that include adropin in the ‘old-old’ brain.

### ENHO expression correlates with aging-related neuropathology

**Fig. 3:**
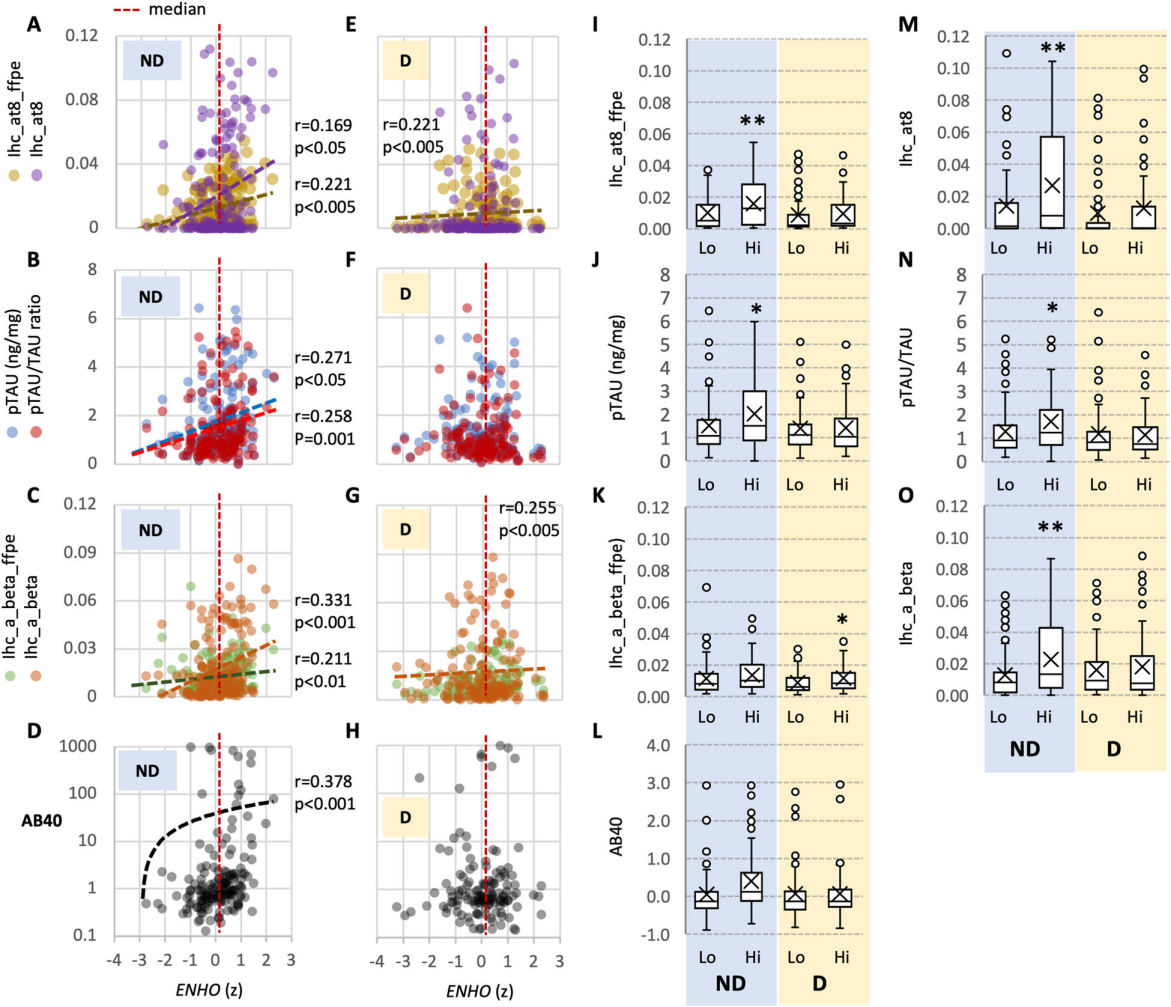
Scatterplots showing relationships between relative ENHO expression (*x*-axis) and protein markers of aging-related neuropathology. Data are shown as scatterplots for samples from study participants with no dementia (**A**–**D**) or with dementia (**E**–**H**). The box-and-whisker plots shown in (**I**-**O**) show the averaged data for D and ND samples split into subgroups (Lo, Hi). ‘Lo’ indicates data from samples with ENHO values below the median (76 samples for D, 90 samples for ND), while ‘Hi’ indicates data from samples with ENHO values above the median (76 samples for D, 90 samples for ND). The markers shown reading top to bottom are AT8 (tau phosphorylation of Ser202 and Thr205), TAU (total phosphorylated tau, and ratio of phosphorylated to non-phosphorylated tau), Aβ, and AB40 (Ab40). Correlation coefficients are shown where associations are significant (*p* < 0.05). For AT8 and Ab staining, data are shown from fresh frozen (ffpe) or paraffin-embedded sections. The data were subdivided into two groups (below or above the median) after being ranked by ENHO expression. The median is shown as the red dashed line in the scatterplots shown in (**A**–**H**). Ranking occurred for all participants (All), or for subgroups separated by dementia status (ND, no dementia; D, dementia). Significantly different from low dementia status, **p < 0.01; *p < 0.05.

**Fig. 4:**
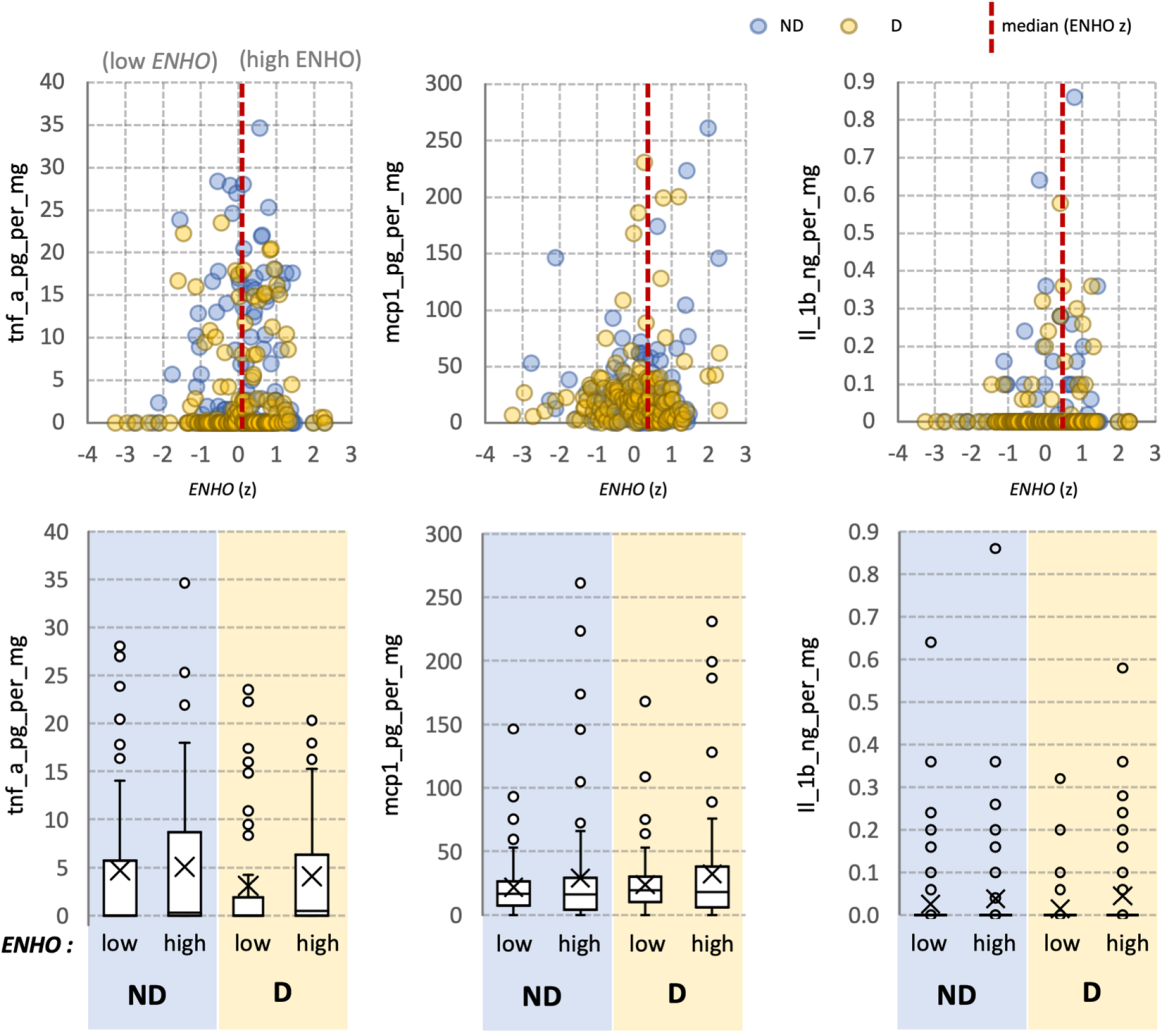
Relationships between *ENHO* expression and protein markers of neuroinflammation in study participants. The top three panels show the data as scatterplots; TNFa, MCP1 or IL1b are plotted against relative *ENHO* expression. The bottom three panels show TNFa, MCP1 or IL1b in participants divided into two groups (below or above the median, labelled “low” or “high”) after being ranked by *ENHO* expression.

The Aging, Dementia and TBI Study includes protein markers of dementia-related pathologies and inflammation by immunohistochemistry (IHC) on fresh frozen and formalin fixed paraffin embedded (FFPE) postmortem tissue samples^48^. In a simple bivariate correlation analysis, levels of *ENHO* transcript correlated positively with tau pathologies (AT8, Aβ plaques, AB40, pTau and pTau/Tau ratio) in the ND group (Fig. 3A–D), but less so in the D group (Fig. 3E–H). These relationships were further investigated by dividing participants into two groups ranked by *ENHO* expression above or below the median (Fig. 3I–O). The groups with high *ENHO* expression appear to exhibit more severe Tau-related pathologies, particularly in participants with no dementia. However, no relationships are observed with inflammatory markers (for example with TNFa, MCP1 and IL1b, Fig. 4). There was however no correlation between *ENHO* expression (averaged for all four structures) and semi-quantitative scores of AD pathology (CERAD, BRAAK, NIA REAGAN) (data not shown).

### Neutrophic properties of adropin in primary cultured hippocampal neurons

**Fig. 5:**
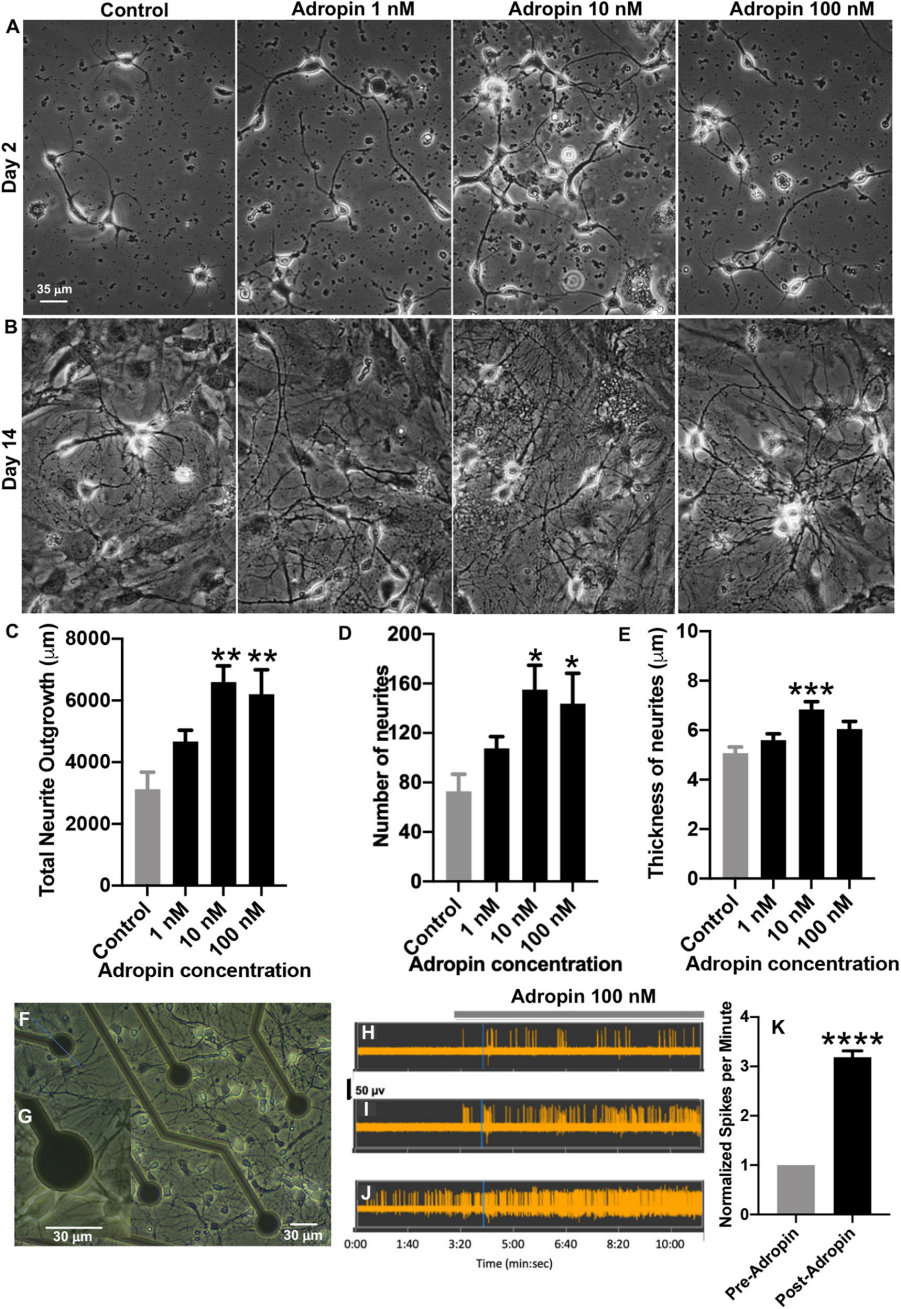
Neurotrophic properties of adropin in primary cultured mouse hippocampal neurons. **A**–**B** Primary cultured hippocampal neurons were cultured in the absence (control) and presence of 1,10 and 100 nM adropin. Phase contrast images were taken at day 2 (early network; **A**) and day 14 (mature network; **B**). **C** Neurons cultured in the presence of 10 and 100 nM adropin for 2 days exhibited longer neurites than neurons without adropin added. The average total neurite outgrowth per phase contrast image of day 2 control neurons was 3125.7 ± 550.5 µm, 1 nM adropin was 4668.3 ± 365.9 µm, 10 nM adroprin was 6599.4 ± 520.7 µm, and 100 nM adropin was 6201.9 ± 791.7 µm (*p* < 0.01 for control vs. 1 nM adropin and control vs. 100 nM adropin; *n* = 8 images analyzed per condition). **D** Addition of 10 nM and 100 nM adropin also significantly increased the number of neuritic branches compared to control neurons. The average number of branches per control image was 72.8 ± 14.0, for 1 nM adropin was 107.5 ± 9.6, for 10 nM adropin was 155.1 ± 19.7, and for 100 nM adropin was 143.8 ± 24.5 (*p* < 0.05 for control vs. 1 nM adropin and control vs. 100 nM adropin; *n* = 8 images analyzed per condition). **E** Neurite thickness was analyzed on neurons cultured for 14 days and showed that 10 nM adropin significantly increased neurite thickness. The average thickness of the five thickest neurites per control image was 5.1 ± 0.3 µm, per 1 nM adropin image was 5.6 ± 0.3 µm, per 10 nM adropin image was 6.8 ± 0.3 µm, and per 100 nM adropin image was 6.0 ± 0.3 µm (*p* < 0.001 for control vs. 1 nM adropin; *n* = 5 thickest neurites measured and averaged in eight images per condition to equal 40 neurites analyzed per condition). **F** Hippocampal neurons cultured on a MEA chip. **G** Higher magnification image showing electrode and neurons. **H**–**J** Sample traces of neuronal spiking activity before and after exposure to adropin (100 nM) using the multi channel recording system. Sample traces demonstrate that adropin increased the spontaneous firing activity in neurons either previously quiescent (**H**, **I**) or active (**J**). **K** Neuronal spiking activity was quantified as “spikes per minute” and normalized to baseline spikes per minute as described in the Methods. Addition of 100 nM adropin significantly increased the spiking frequency of the neurons. Normalized spikes per minute after 100 nM adropin was 3.2 ± 0.1 spikes per minute (*p* < 0.0001; *n* = 31 electrodes). Data are presented as mean ± S.E.M. * indicates *p* < 0.5, ** indicates *p* < 0.01, *** indicates *p* < 0.001, and **** indicated *p* < 0.0001.

To investigate whether variation in adropin expression has causal effects on neurological aging we used primary culture cells and mouse models. Adropin^34-76^ exhibits dose-dependent trophic properties in primary cultured mouse hippocampal neurons (Fig. 5A–G). Phase contrast images taken on day 2 (Fig. 5A) and day 14 (Fig. 5B) and neurite tracing analyses using ImageJ neurite tracing methods^60–62^ revealed adropin^34-76^ significantly promoted neuritogenesis (*p* < 0.001) (Fig. 5C). The number and thickness of branches in neurons were also significantly increased (*p* < 0.05) (Fig. 5D, E).

To determine if adropin regulates neuronal excitability, hippocampal neurons were cultured on the non-invasive MEA for recording of neuronal network activity (Fig. 5F, G). After 9 days of culture, adropin (100 nM) was added and neuronal activity recorded before (baseline) and after treatment. The frequency of neuronal activity was significantly increased in hippocampal neurons regardless of baseline activity. Hippocampal neurons that were quiescent at baseline (Fig. 5H–I) began firing action potentials after treatment. In neurons showing spontaneous activity, application of adropin significantly increased firing frequency (spikes/min; Fig. 5J). Statistical data for the recordings (Fig. 5K) demonstrated a neuroexcitatory effect of adropin on hippocampal neurons.

### Adropin expression in the C57BL/6J (B6) mouse brain

**Fig. 6:**
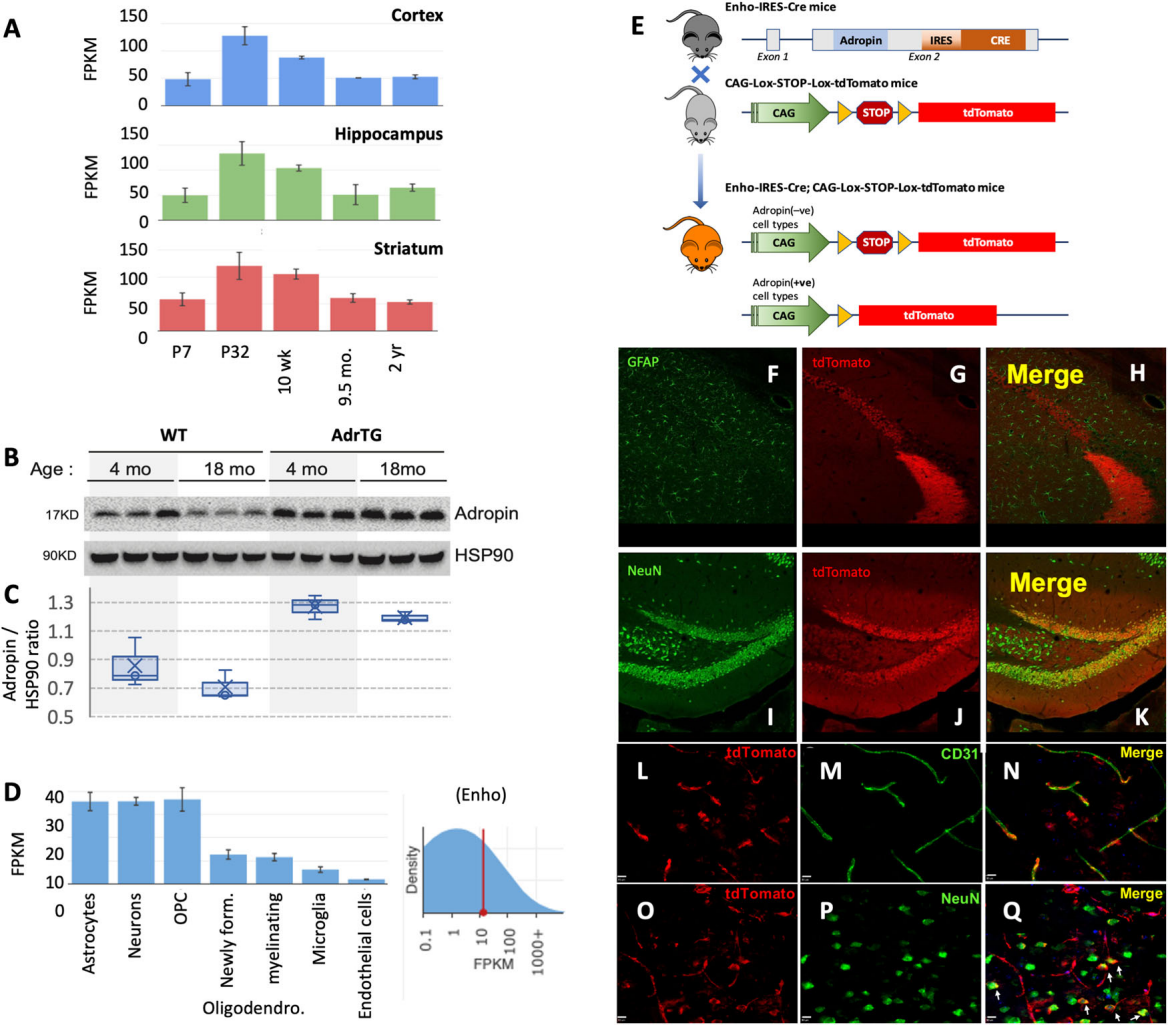
Adropin expression in the mouse brain. **A** Expression profiling in mouse brain structures by age show a peak at postnatal day 32 (P32). **B**, **C** Adropin immunoreactivity in brain lysates of AdrTG mice compared to wild type (WT) littermates at 4 and 18 months of age. WT mice show a decline in adropin protein levels at 18 months that is not observed in AdrTG. HSP90 was used as a loading control. **D** Profiling of *Enho* expression in isolated cells from the mouse brain. **E** The top panel is a schematic of the approach used to generate an ENHO-Cre reporter mouse. The lower panels (**F**–**Q**) are representative photomicrographs from hippocampal sections (**F**–**K**) and high magnification photomicrographs of vessels in the cortex (**L**–**Q**). Scale bars (10 μM) are shown in the bottom left in (**L**–**Q**).

In Sprague Dawley rats adropin protein levels in plasma and brain extracts are lower in 18-month old relative to 4-month old animals^40^. Here we report that *Ehno* expression in the peaks between the 1st and 5th week of life in the cortex, HIP and striatum (Fig. 6A)^63^. Adropin protein levels determined by western blot using brain samples are also lower in 18-month-old B6 mice compared to 4-month-old animals (Fig. 6B, C).

Open access data suggest less cell-type specificity in *ENHO* expression in cells isolated from the mouse brain compared to humans (Fig. 6D)^64^. High expression is observed in astrocytes, neurons and oligodendrocyte precursor cells. Lower expression is observed in newly formed and myelinating oligodendrocytes, and in microglial/macrophages. Low expression is also observed in endothelial cells (Fig. 6D)^64^. Cell-specific patterns of *Enho* expression were further investigated using B6 mice with IRES-Cre inserted into the *Enho* locus crossed with the CAG-lox-STOP-lox-tdTomato reporter strain (Fig. 6E). Cre-induced expression of tdTomato was observed throughout the mouse brain (Fig. 6F–Q). In the mouse HIP expression was also observed in neurons, but surprisingly not in astrocytes (Fig. 6F–Q). Expression was also observed in cortical neurons and endothelial (CD31^+ve^) cells (Fig. 6L–Q), in agreement with experiments using mouse and human endothelial cell lines^20,59,65^.

Genomic data from the aged human brain indicates inverse associations between the expression of adropin and genes involved in mitochondrial, neurotrophic, and inflammatory processes. These relationships were investigated in the mouse brain using data from open access transcriptome databases and mouse experiments performed at the Saint Louis University.

**Fig. 7:**
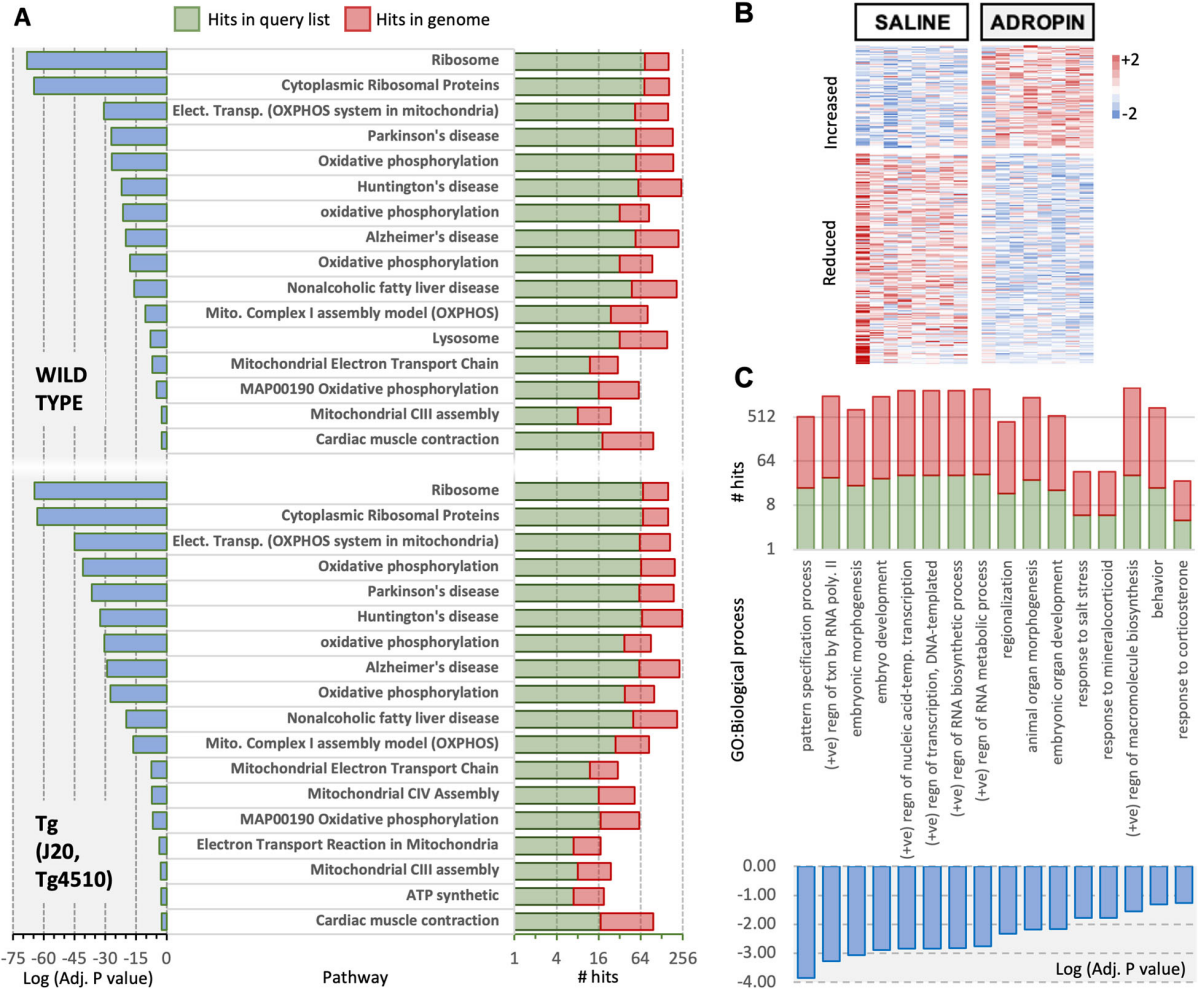
Adropin effects on the transcriptome of the aged mouse brain. **A** Gene enrichment analysis of genes correlating with the *ENHO* transcript in wild type (*n* = 63) or transgenic mouse models used in Alzheimer’s disease (*n* = 58) (GSE125957). Pathways correlated with ENHO expression were similar between genotypes and are related to mitochondrial processes and synthesis of large macromolecules. **B** Heat map showing genes increased or reduced following adropin treatment of 18-month-old male B6 mice with saline or adropin^34-76^ for 1 month (*n* = 8/group). These mice were selected from the animals used in the behavioral studies shown in Fig. 8. **C** Results from gene enrichment analysis showing biological processes responding to adropin^34-76^ treatment.

We first analyzed RNA seq data from experiments involving two lines of transgenic mice used in the investigation of Alzheimer’s disease (GSE125957)^66^. These transgenic lines overexpress either a human mutant of tau (P301L) or amyloid precursor protein (K670N/M671L and V717F). *Enho* expression was not identified as a differentially expressed transcript between genotypes^66^. However, gene enrichment analysis using an *r* > 0.7 as the selection criteria indicates that *Enho* expression correlates with pathways also observed in humans (Fig. 7A). GO:Molecular Function terms overlapped between the two populations and were related to protein translation (for e.g., “Ribosome”, “Cytosolic Ribosomal Proteins”) and mitochondria (“oxidative phosphorylation”, “Electron Transport Chain”). *Enho* expression also correlated with pathways associated with neurodegenerative diseases (“Parkinson’s disease”, “Alzheimer’s disease” and “Huntington’s disease). It is also noteworthy that the strength of the correlations, particularly those related to mitochondrial function, were stronger in transgenic strains expressing mutant proteins causing LOAD in humans (Fig. 7A).

The positive correlations between *Enho* and either *Apoe* or *Clu* observed in humans appears to be retained in the mouse brain (Fig. S3), although only *Clu* meets the selection criteria (*r* > 0.7). However, genes involved in insulin signaling did not show the same relationship (Fig. S3). Moreover, astrocyte markers were not enriched in the genes correlating with *Enho* in the mouse brain. This result is consistent with a less cell-specificity in the mouse nervous system (Fig. 6D).

### Effects of adropin treatment on the aged mouse brain transcriptome

Transgenic mice over expressing adropin^1-76^ (AdrTG) under the control of a human b-actin promoter^14,17,30,41^ were used investigate whether variation of adropin expression affects genes in pathways correlating with ENHO in the human brain. The mouse model used for the experiment (male B6 mice aged 18 months) was selected based on the development of cognitive impairment at or around this age which corresponds to a human age of 56–69 y^67^.

**Fig. 8:**
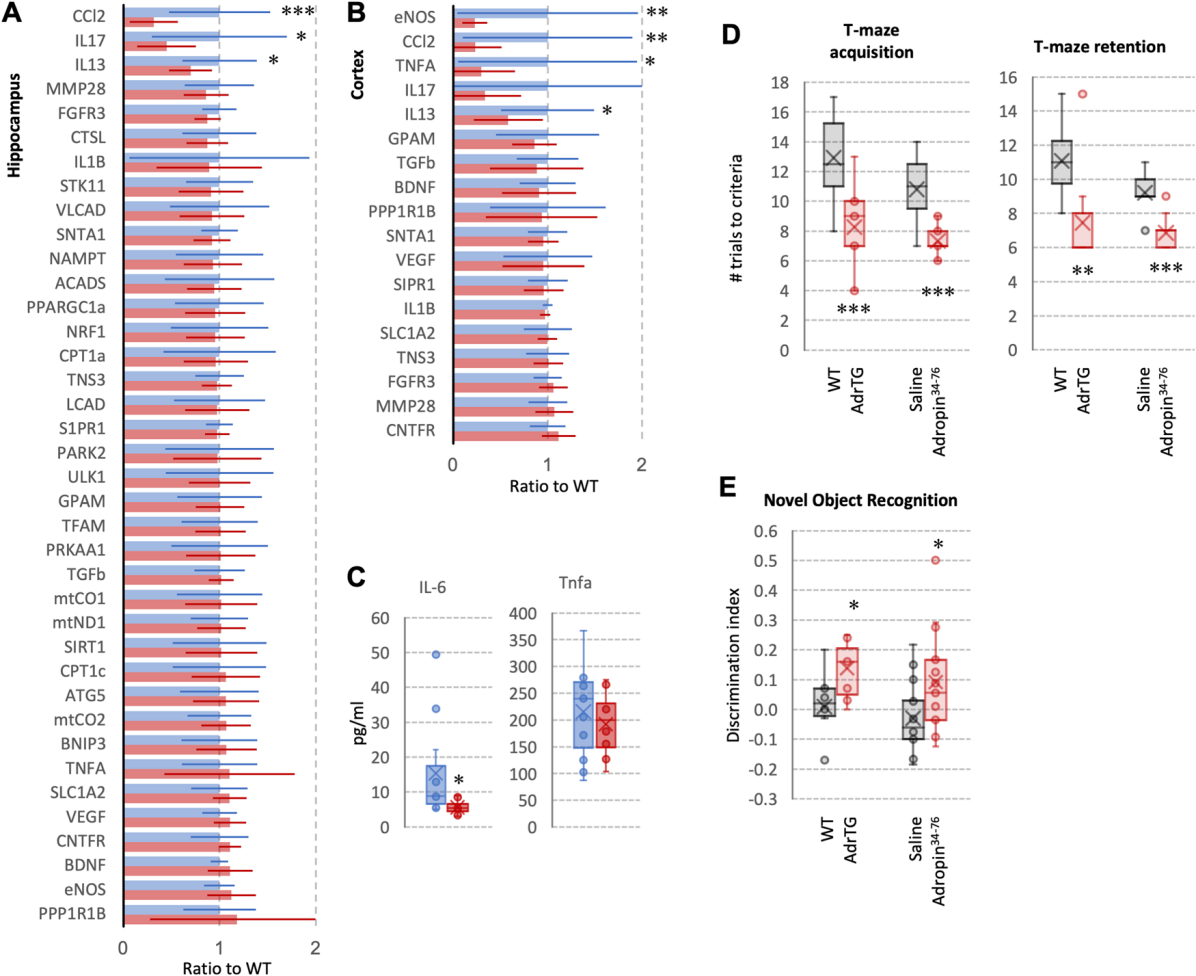
Reduced expression of genes involved in inflammation and improved performance in tests of spatial learning and memory and novel object recognition in 18-month-old mice treated with adropin. **A**, **B** Reduced expression of genes involved in inflammation in the hippocampus (**A**) and cortex (**B**) of male 18-month-old AdrTG mice (*n* = 13) compared to age-matched controls (*n* = 12). Expression data (mean, SD) reported as a ratio of WT controls for each gene against 3 ‘housekeeping’ reference genes (*Hprt1, 36b4, Ppib*). **C** Serum levels of IL-6 but not TNFA are lower in AdrTG compared to controls. Significance is indicated by *(*p* < 0.05), **(*p* < 0.01), or ***(*p* < 0.001). **D**–**E** Improved performance in tests of spatial learning and memory (aversive T-maze) (**D**) or novel object recognition (**E**) of 18-month-old AdrTG (*n* = 9) or B6 mice treated with adropin^34-76^ for 2 weeks (*n* = 14) compared to the respective age-matched control group (WT littermates for AdrTG, *n* = 10; mice treated with diluent for the adropin^34-76^ treatment group; *n* = 15). Significance is indicated by *(*p* < 0.05), **(*p* < 0.01), or ***(*p* < 0.001).

Increased adropin protein levels are observed in brain lysates from AdrTG mice compared to WT controls, particularly at 18-months of age (Fig. 6B). The results from analysing gene expression using qRT-PCR suggested that adropin protects against neuroinflammation (Fig. 8A–C). Specifically, the expression of genes involved in inflammation were lower in the HIP (Fig. 8A) and the cortex (Fig. 8B) of AdrTG compared to age-matched controls. Measurement of serum cytokines indicated a significant difference (*p* < 0.05) in IL-6 concentrations, with 60% lower levels in AdrTG mice (Fig. 8C). However, circulating levels of TNFA (Fig. 8C) and other cytokines were not significantly different (Fig. S4). We also assessed for tau phosphorylation and markers of oxidative stress. However, the expression of these markers was highly variable and mostly below the limits of detection (data not shown), likely explained by the mild model used for the study (18-month-old male B6 mice). The expression of genes involved in other biological processes were also not significantly different in AdrTG (Fig. 7A, B).

The putative secreted domain (adropin^34-76^) is sufficient for biological activity when administered by ip. injection to mice, inducing changes in mitochondrial fuel selection of skeletal muscle^14,17–19,30^. To investigate whether adropin^34-76^ administered also regulates the brain transcriptome, RNA seq was used in 18-month-old male B6 mice treated with synthetic peptide at a dose previously shown to be effective. Mice were split into two weight matched groups (Fig. S5A), acclimated to handling and then treated with adropin^34-76^ (90 nmol/kg/d) suspended in 0.9% saline plus 0.1% BSA over 4 weeks. Controls were treated with diluent; injections were given daily at 0900 h. Applying a cut-off using a fold-change > 1.5 and a *p* value of <0.05 identified a small number of responsive genes (84 upregulated, 172 downregulated) (Fig. 7B). Gene enrichment analysis indicated that adropin^34-76^ treatment primarily changed the expression of genes involved in tissue morphogenesis (e.g., “pattern specification process”, “embryo development”, “animal morphogenesis”) (Fig. 7C). Possibly related to increases in cellular proliferation and differentiation, there was also evidence for increases in large molecule synthesis. For example, RNA metabolism (“positive regulation of RNA biosynthetic process”; “positive regulation of transcription by RNA polymerase II”) and ‘macromolecule synthesis” were affected by treatment. Less significant but still noteworthy were pathways related to behavior and stress responses. The only pathway showing a significant enrichment was the “AP-1 transcription factor network” that includes genes responsive to external stimuli (*Fos*, *Fosb*, *Dusp1*, *Junb*, *Th*, *Ccn1* and *Agt*). GO:Molecular Function terms were mostly related to transcription factor activity and RNA transcription, which is consistent with the correlations between ENHO expression and ribosomal genes in open access transcriptome data (Fig. 7A).

### Adropin improves spatial learning and memory in 18-month-old B6 mice

Tests of spatial learning and memory and novel object recognition (NOR) were used to determine whether adropin has a positive effect on cognitive function in 18-month-old male B6 mice. Male AdrTG mice performed significantly better in tests of spatial learning and memory, and of recognition memory compared to age-matched WT controls (Fig. 8D–E). Treatment with adropin^34-76^ similarly improved performance in trials (Fig. 8D–E). Fasting glucose and insulin data were recorded at the completion of the study; no significant treatment effects were observed for either parameter suggesting that the behavioral effects are independent of marked improvements in glycemic control (Fig. S5B). Collectively, these results indicate that high expression of adropin may protect against mild cognitive decline with aging, and that acute treatment may reverse the effects of aging.

### AdrTG mice exhibit improved resilience in tests of energy balance and ambulatory activity

We next compared how adropin over expression affects aging-related changes of whole-body energy balance and ambulatory movement using mice either ‘middle aged’ (M, 8–10 mo) or ‘old’ (O, 16–20 mo.). The respiratory exchange ratio (RER, VCO_2_/VO_2_) provides an estimate of whole body substrate selection^68^. The RER increases during the dark period as chow-fed mice move to oxidizing ingested carbohydrates and relying less on mobilized fatty acid (Fig. S6A, B). WT-O mice exhibited low RER, indicating increased dependency on fat reserves (Fig. S6A). In females, the RER transition between light and dark periods was more pronounced in AdrTG, irrespective of age (Fig. S6B). Oxygen consumption exhibited the diurnal rhythm predicted from increased ambulatory activity and food intake during the dark period (Fig. S6C, D). This feature of energy metabolism was not markedly affected by genotype.

Food intake and changes in body weight during the recordings were consistent with RER data, indicating improved maintenance of body weight in M and old-AdrTG mice. Male WT-O mice lost weight during the recording (mean ± SD and *n* for weight gain/loss in g, young-WT −0.1 ± 0.2, *n* = 4; old-WT −5.8 ± 3.4, *n* = 3; young-AdrTG −0.4 ± 1.0, *n* = 4; old-AdrTG −1.7 ± 0.5, *n* = 3). Female WT mice also tended to lose more weight (mean ± SD for weight gain/loss in g, young-WT −3.0 ± 2.5; old-WT −1.6 ± 2.9; young-AdrTG −0.1 ± 0.5; old-AdrTG −0.6 ± 0.5, *n* = 4 all groups). Weight loss correlated with lower food intake during the recording period (Fig. S6E). Pooling data from male and female mice indicated WT mice were more prone to weight loss during recordings (−2.4 ± 3.0 vs. −0.6 ± 0.8, *p* < 0.05). Advanced age may exacerbate the phenotype (Fig. S6F).

Ambulatory movement exhibited the predicted diurnal profile, irrespective of age or sex or genotype (Fig. S6G, H). Aging of WT mice, but not in AdrTG mice, appeared to associate with reduced movement (Fig. S6G-I). A negative energy balance indicated by low food intake and weight loss might be predicted to account for this phenotype. Food intake is a strong predictor of weight gain/loss (Fig. S7A). However, it is not a strong predictor of ambulatory movement (Fig. S7B). Weight gain/loss is also only a weak predictor of ambulatory movement (Fig. S7C). AdrTG aged < 6 months exhibit a lean phenotype and improved glycemic control^14^. At 18 months of age, evidence for modest differences in nutrient partitioning that suggest a lean phenotype are still observed (Fig. S8A-F), however glucose clearance is normal (Fig. S8G).

Collectively, these results indicate significant genotype effects on feeding behavior, substrate selection glucose vs. fat oxidation and ambulatory movement. As prolonged anorexia is not compatible with survival, superior adaptation of AdrTG to the stress of being moved to a novel environment (metabolic caging) is a plausible explanation for the behavioral and metabolic phenotypes.

## Discussion

These results indicate that adropin functions support brain health during aging. In the human and mouse brain transcriptome, expression of the *ENHO* transcript encoding adropin correlates positively with gene networks involved in mitochondrial energy metabolism and the synthesis of macromolecules supporting cellular functions. The brain accounts for 20% of the body’s energy consumption, with glucose being the primary fuel source^69^. It is therefore tempting to speculate that adropin expression in the brain will positively correlate with glucose uptake and utilization. Certainly, the positive correlation with processes involved in macromolecule synthesis and RNA metabolism suggests expression associates positively with metabolic activity at a cellular level.

The inverse associations with AKT3 and GSK3B in the human brain indicate this interpretation is perhaps overly simplistic. AKT3 belongs to a family of serine/threonine kinases (AKT1–3), and regulates brain development and cognitive function^70^. Phosphorylation of GSK3 by AKT3 may also affect behavior^70^. Given the roles of GSK3 in mediating tau hyperphosphorylation^47^ and affecting behavior, the inverse association with GSK3B is also potentially of interest. Further experiments investigating the regulation of glucose metabolism in astrocytes and neurons, and of the relationships between adropin and GSK3 activity in the brain, are clearly needed.

Peak adropin expression in the first decade of life suggests adropin has a role in regulating developmental processes. This hypothesis is supported by the phenotype of adropin knockout mice which display deficits in synapse formation, coupled with decreased locomotor activity and impaired motor coordination^31^. We also show that adropin at physiological concentrations acts directly on neurons, significantly enhancing the number, length, and thickness of neurites in the early development of cultured neurons (within first 2 days in culture). Increased thickness of primary neurites correlates with increased neuronal surface area for housing ionic channels/receptors, promoting efficiency of neuronal conductivity and synaptic transmission. In addition, adropin significantly increases neuronal activity in primary hippocampal neurons. Together, the neuritogenic and neuroexcitatory effects of adropin on hippocampal neurons may contribute to its function in promoting high cognition in animals and humans.

Growth and development of the human brain requires an abundance of calorie-dense diets, including essential fatty acids to provide energy and substrates for cell division, cellular morphogenesis, and synaptic functions^71^. It is therefore interesting to note the positive correlations between circulating adropin levels and selection of diets with high energy content^72,73^. Nutrient-sensing mechanisms have been identified that drive macronutrient preferences to match macronutrients selection with energy requirements^74,75^. While speculative, preference for high fat diets in people with high adropin levels could correlate with increased nutrient demand by the nervous system. Indeed, in young people plasma adropin concentrations appear to correlate positively with a lean phenotype^41^. Further investigation of the relationships between adropin expression, plasma adropin concentrations, and nutrient requirements of the brain are clearly needed.

The relationships between *ENHO* and the expression of candidate genes (*APOE*, CLU/ApoJ) and pathways involved in cholesterol metabolism in the brain are relevant to aging. APOE has a critical role in mediating cholesterol transport in the brain^43^. CLU/ApoJ has also been implicated in lipid metabolism, and functions as extracellular chaperone linked to Aβ clearance and toxicity^45^. The negative relationship between ENHO expression and “REACTOME_CHOLESTEROL_BIOSYNTHESIS” is also consistent with our recent studies showing relationships between adropin and cholesterol metabolism. In humans and NHPs we have observed an inverse correlation between adropin and plasma markers of cholesterol metabolism^28,41^. It is reasonable to speculate that high adropin expression in the brain could increase plasma concentrations of adropin peptide, and correlate with brain cholesterol metabolism. Plasma adropin concentrations in humans are higher in males compared to females, particularly early in life, and decline with aging^41^. Based on gene expression analysis, neural tissues appear to be a plausible site of origin for circulating adropin in humans. It will be important to determine whether adropin protein levels correlate with gene expression. Expression of the mature *Enho* mRNA correlates with levels detected in ribosomal fractions in the mouse liver. Whether this relationship is also observed in the nervous system needs further study.

The conservation of the correlations between adropin and metabolic pathways between mice and humans suggests that the responses of mice to treatment could be translatable. It is important to note that the brain samples in the clinical studies came from individuals who died of natural causes or by accident. In contrast, the mice were euthanized at specific ages and times. This latter point is potentially important, as ENHO expression in the NHP brain exhibits a circadian profile^28^.

In humans, advanced age appears to result in changes in the pathways correlating with *ENHO* expression. Correlations observed in those with advanced age are also dependent on dementia status. In the ‘old-old’ without dementia, correlations develop with gene networks involved in morphogenesis, including the growth of axonal and synaptic processes. Adropin has been implicated in brain development in mice^31^, and here we observed potent effects of synthetic adropin on development of neuronal processes in cultured neurons, indicating a direct effect. This could suggest adropin signaling maintains neuronal processes critically involved in cognitive function during aging.

In ‘old-old’ people diagnosed with dementia, the *ENHO* transcript clustered with gene networks involved in mitochondrial energy metabolism, with terms related to vascular function also appearing in the analysis. Adropin has been shown to regulate differentiation of human umbilical vein endothelial cells into capillary-like structures, while transgenic over expression of adropin stimulated angiogenesis and improved blood flow in a mouse model of ischemia^20^. Clinical studies have observed positive correlations between circulating adropin levels and endothelial function^57,76^. Actions of adropin on vascular function could be relevant to the aging brain, as reduced cerebrovascular blood flow has been linked to aging-related cognitive impairment and dementia^77^.

The results from studies using aged mice provide further indication of high adropin expression benefiting cognitive performance. Furthermore, providing synthetic adropin as a supplement to aged mice in which adropin protein levels have declined appears to have a therapeutic effect. However, the positive correlations between *ENHO* expression and protein markers of tau pathology contradict this conclusion. There is considerable variation in signatures of brain inflammation; these markers do not always correlate with dementia status in the ‘old-old’ cohort used for the current study^48^.

It is difficult to interpret the differences between the correlative data observed in postmortem samples from people aged from birth to 40 years (mature adults) and the ‘old-old’. Reduced fidelity in transcriptional and posttranscriptional mechanisms affecting transcript abundance is a plausible explanation for lower correlation coefficients. However, survivorship bias should be considered when comparing differences in pathways and biological processes correlating with the *ENHO* transcript in the D and ND groups. Transitioning to correlations with neural morphogenesis could indicate a survival advantage in the ‘old-old’ without dementia. A similar reasoning could be applied to the retention of mitochondrial processes and transitioning to associations with vascular function in the ‘old-old’ with dementia. This does not necessarily imply that high levels of adropin expression per se confers a survival benefit. Rather, retaining regulatory control of gene networks that include the *ENHO* gene may act in concert to confer a survival advantage in the old-old. On the other hand, the data from mouse studies clearly imply that high adropin expression may be beneficial.

Similarly, the trend for higher *ENHO* expression in the ‘old-old’ carriers of the APOE ε4 allele could indicate survivor bias. GWAS have linked APOE gene variants to longevity^78^. Inheritance of the ε4 allele increases risk of early mortality from cardiovascular disease and LOAD. That *ENHO* expression tends to be higher in people aged over 75 y with >0 APOE e4 alleles could indicate a selection advantage. In this scenario, a combination of the APOE e4 allele with high adropin expression delays the development of LOAD. An alternative interpretation is that inheritance of APOE ε4 allele associates with cellular stress phenotypes in the nervous system that increase *ENHO* expression. For example, increases in *Enho* expression in the mouse live have been linked to oxidative stress^30^. The two theories are also not mutually exclusive, with activation of adropin expression and signaling in response to stress having a protective effect.

Aging-related transitions in gene networks that correlate with *ENHO* could also indicate responses to changes in the cellular environment. Cellular stress responses driving age-related inflammation could dominate, thereby altering the regulatory framework driving the transcriptional and posttranscriptional processes affecting the levels of *ENHO* and other transcripts. The observation that the gene networks correlating with *ENHO* differ between the ‘old-old’ diagnosed with dementia (mostly LOAD) and those who died with normal cognitive function is consistent with this theory.

It is important to note some of the limitations of the human studies. For example, differences in nutrition and environment during childhood between participants of the GTEx and the Aging, Dementia and TBI Study could be important. The participants in the latter study were born in the early 20th Century when obesity and type 2 diabetes were less prevalent. On the other hand, postmortem samples in the GTEx portal come from people living in an obesogenic environment.

Pending clinical studies designed to administer adropin to people with advanced age, the human data reported here are by necessity correlative. Mouse models were used to investigate whether higher adropin expression confers an advantage in aging, and also whether administration of synthetic peptide would be beneficial.

The AdrTG mice used for these studies exhibit increased adropin protein expression in the brain. However, the use of a ubiquitous promoter increases expression in all tissues^14,17,30,41^. The results from experiments using 18-month-old male mice nevertheless suggest increasing expression of adropin improves cognitive function. Cognitive function was assessed by tests of spatial learning and memory and NOR. The use of mice over expressing adropin throughout the lifespan complicates the interpretation of these results. However, injections of adropin peptide improved cognitive performance in 18-month-old B6 mice. The effects of adropin to improve cognitive performance can thus be dissociated from metabolic phenotypes earlier in life. Moreover, the results from this acute treatment experiment suggest that treatment with synthetic adropin can reverse aging-related cognitive impairment.

The results from assessing whole body energy balance could be interpreted as indicating increased resilience in response to a novel environment. Old mice transferred to metabolic cages exhibit anorexia, reduced activity, weight loss and mobilization of fatty acids. This response was not evident in age-matched AdrTG. Future studies examining how adropin over expression affect behavioral flexibility and stress responses in aging are clearly warranted. While technically challenging, behavioral studies using ‘old-old’ mice aged >2 years could also be informative.

A weakness with the mouse studies is the lack of apparent clear mechanism explaining the phenotype. Gene expression analysis suggests reduced inflammation and neurotrophic mechanism as potential factors. Other mechanisms based on the known functions of adropin are possible that include effects on blood flow^20^ or the blood brain barrier^59^. Direct neurotrophic actions on neurons were also observed. However, it is currently not known whether adropin^34-76^ administered ip. penetrates the blood brain barrier.

The current results nevertheless provide an indication that high expression during the later stages of life, or administration of the synthetic peptide or derivative thereof, confer a benefit. Further experiments exploring mechanism(s) are clearly indicated. Whether adropin expression correlates with lifespan, and whether over expression is effective in mouse models of severe LOAD, also needs to be explored.

In summary, current demographic trends indicate that the burden of dementia will increase this century as the population ages. The current study has applied a translational approach to identify a novel candidate for developing therapies to improve cognitive function in aging. Further investigation of the adropin neuropeptide with respect to cognitive function and brain health are clearly warranted.

## Methods

### Analysis of transcriptome data

For pre-processing of The Aging, Dementia and TBI Study Patient, datasets were downloaded from https://aging.brain-map.org/download/index. The data included: (i) de-identified clinical information for 107 patient donors, (ii) normalized gene-level FPKM (Fragments Per Kilobase of transcript per Million mapped reads) expression for 377 unique samples obtained from these donors, (iii) A sample metadata table and (iv) a gene ID and gene symbol table for 50,281 unique genes. To determine whether the samples were normalized, we plotted the density of histograms for all samples. To perform the correlation analysis, we pre-processed the expression matrix using “log2(FPKM+1)”. All analyses were performed using Bioconductor^79,80^ in R.

GSEA (Genome-wide gene set enrichment analysis) used a scaled correlation matrix to reveal *ENHO*-coregulated gene pathways^81^. GSEA is used to evaluate the enrichment or depletion of a given gene set/pathway relative to one of the two functional states, which are distantly connected by a gradient of genes pre-ranked based on a measurable activity. Typically, these are differentially expressed genes. In our analysis, we defined the two states as, (i) a gene state positively associated with ENHO expression, and (ii) a gene state antagonistic or incompatible with ENHO expression.

To perform GSEA, 5529 experimentally-verified biological pathways were downloaded from the Molecular Signature Database^82^ (V7.1 C2) (https://www.gsea-msigdb.org/gsea/msigdb/index.jsp). Pearson Correlation coefficients were calculated between ENHO and each of the 50281 genes. This was done using the entire dataset containing all 377 samples or only a portion of them with specific features (Dementia/No Dementia, Male/Female or both). The correlation coefficients were then ranked, centered and scaled. The resulting pre-ranked correlation coefficients were used to search for enriched or depleted pathways from the 5529 experimentally-verified biological pathways using the “fgsea” package in R. Genes not included in the MSigDB pathways were removed from the list of pre-ranked genes. Significant pathways with an adjusted *p* value ≤ 0.05 were selected for further analyses. Top 20 enriched or depleted pathways were plotted. These significant pathways were saved into excel files.

For the other datasets referenced in the Result sections, data were downloaded and log transformed if needed. Genes were ranked by the correlation coefficient determined using Microsoft Excel. Functional enrichment analysis used the ToppFun tool (https://toppgene.cchmc.org). Biological processes in separate populations (D, ND, age < 40 y) were assessed using the PercaiAI CompBio platform; the Assertion Engine was then used to determine conservation of pathways between populations. Each Assertion produces similarity scores from 0 to 1, with scores >0.1 indicating substantial overlap. Outputs of Assertion Engine queries are heatmaps that show overlap scores between datasets.

### Neuronal cell culture, phase contrast imaging, and ImageJ neurite tracing analysis

Hippocampal neurons were dissected from B6 mice and cultured from newborn pups (postnatal day 2, P2) as described previously^83,84^. Briefly, hippocampal tissue was isolated and single cells were dissociated with papain (50 µg/mL). Single-cell suspensions were created via trituration with pipettes of decreasing size. The cells were diluted in culture media (neurobasal medium, 2% B27, L-Glutamine [200 mM], 4% FBS, and penicillin-streptomycin [Invitrogen]) and plated on dishes coated with poly-D-lysine (100 µg/mL) and laminin (2 µg/mL). For phase-contrast imaging, cells were cultured on glass-bottom petri dishes. For neuronal activity recording, primary cultured neurons were cultured on Multi-electrode array (MEA) Neurochip (Multi Channel Systems) was coated with poly-D-lysine and laminin, and cells were plated directly on the Neurochip. Hippocampal neurons were kept at 37 °C in an airtight modular incubator chamber (Thermo-Forma) circulated with 5% carbon dioxide and medical air. Every 3–4 days, half of the culture media was replaced with fresh media.

To examine the effect of adropin on the formation of early networks, culture media supplemented with 1 nM, 10 nM, or 100 nM adropin was added to culture dishes on the same day as plating (day 0). We counted and measured the number of branches and total neurite outgrowth (measured in each phase contrast image taken) on day 2 and the neuritic thickness of primary neurites (processes derived directly from cell bodies) on day 14. The number and length of neurites in day 2 and the primary neurites in day 14 cultures are easily discernible, and hence we chose to analyze these parameters. Quantitative data and statistical analyses are shown in Fig. 5. Hippocampal neurons grew for 2 days (day 2), and phase contrast images were taken with an inverted microscope (Olympus CKX53). To examine adropin effects on developed neuronal networks, primary hippocampal neurons were grown for 9 days in normal neurobasal culture media. On day 9, 1 nM, 10 nM, or 100 nM adropin was added to culture media and phase contrast images were taken 5 days later (day 14). In all phase contrast experiments, control dishes received no adropin; microscope parameters were the same for control and treatment dishes.

ImageJ was used to analyze phase contrast images of primary hippocampal cells. The ImageJ plugin NeuronJ was employed to measure neurite characteristics in phase contrast images of hippocampal neurons 2 or 14 days in culture in control (no adropin added) and adropin- (1, 10, and 100 nM) added culture as previously described^61,62,84,85^. NeuronJ was programmed to output total neurite outgrowth (µm) and the number of neuritic branches for day 2 cultures and the thickness of primary neurites in day 14 cultures. This is because in day 2 cultures, the length and number of neurites from each cell can be easily identified and traced using ImageJ. For cells that were maintained in culture for 14 days, it is hard to judge the terminal end of each neurite, as extensive networks are formed and glial cells are proliferated and formed layers underneath. We, therefore, focused on analyzing the thickest primary neurites in day 14 cultures. In addition, for day 2 neuritic outgrowth and branching analyses, all neurites within a phase contrast image were analyzed. For day 14 neuritic thickness analysis, the five thickest primary neurites were measured in each image.

### Multi-electrode array (MEA) recording of neuronal activity and spiking frequency analysis

The MEA2100-System Neurochip consists of 60 electrodes organized in an 8*8 grid, designed to capture neuronal electrical activity (Multi Channel Systems). Hippocampal neurons were cultured on the Neurochip as described above and grown for nine days (day 9) to allow for synapse formation. Neuronal activity was measured with the MEA2100-Lite head stage connected to the MCS-IFB interface board. The baseline of neuronal activity was first recorded for 4 min or until the neuronal activity among majority of electrodes was stabilized to act as a pre-treatment baseline. After 4 min, 100 nM adropin was added to the medium, and electrical activity recordings were taken for 8 more minutes by the Multichannel Experimenter software. Neuronal activity was quantified as “spikes per minute,” the number of neuronal firings that occurred within 60 s of recording. Spikes per minute (pre- and post-adropin addition) was normalized to baseline spikes per minute (pre-adropin) for each electrode. This allowed a fair comparison between all electrodes, whether they were previously quiescent or actively firing during baseline recordings.

### Animal studies

Studies involving mice were conducted with the approval of the Institutional Animal Care and Use Committees at the Saint Louis University School of Medicine and the University of Florida. Sentinels were tested regularly to ensure our facility is virus- and pathogen- free. Food and water were available on an ad libitum basis and the rooms had a 12 h light-dark cycle with lights on at 0600 h.

AdrTG were generated and maintained as previously described^14^. For aging studies, mice were maintained in group housing (3–4/cage) with ad libitum access to water and standard rodent chow. For the experiment investigating the response of aging mice to adropin peptide, male B6 mice were purchased at 18 months of age from Jackson Laboratories (Bar Harbor, ME). Mice were acclimated to local conditions for 2 week and then divided into two weight matched groups (*n* = 15/group). The mice were treated with 90 nmol/kg/d adropin^34-76^ (ABClonal Science, 98.66% purity) suspended in 0.9% sterile saline with 0.1% BSA. The peptide was resuspended daily prior to injection and administered as a single ip. injection (0.1 ml volume) at 0900 h. Controls received saline diluent only. Mice were weighed daily; after 2 weeks of injections, mice were subjected to behavioral testing. Treatment continued during behavioral testing.

At completion, mice were fasted for 6 h. Blood glucose (tail snip) levels were recorded using a glucometer; blood samples were collected for analysis of measurement of insulin by ELISA (Ultra Sensitive Mouse Insulin ELISA kit, Crystal Chem USA, IL, USA). Brain tissue samples were collected and snap frozen using liquid nitrogen. Fasting glucose and glucose clearance were assessed in mice fasted for 6 h. Glucose tolerance tests were performed using 1 mg/kg dextrose administered intraperitoneally (ip.), as previously described^30^.

To visualize cells expressing adropin, an IRES-Cre was inserted into the 3′untranslated region (UTR) of the adropin open reading frame in exon 2. The targeting vector was constructed using recombineering system. Isogenic DNA containing the Enho locus was retrieved from genomic colony RP23–100C7 of C57Bl/6 BAC genomic library via gap repair. An IRES-Cre-Frt-neo-Frt was inserted into 3′ 53 bp downstream of the translational stop codon in exon 2. For gene targeting, 50 μg of linearized targeting vector consisting of 3.5 kb 5′arm and 7.2 kb 3′ arm was electroporated into Bruce4 B6 embryonic stem (ES) cells. Homologous recombination in targeted clones was confirmed with Fidelity PCR at the 5′ and 3′ ends. The fragments produced from Fidelity PCR with these primers were sequenced to verify the correctness of recombination.

Correctly targeted ES cells were injected into Albino B6 blastocysts; germline transmitting chimeric mice were obtained and mated with Albino B6 mice to generate heterozygous carriers of the EnhoIRES-Cre-Frt-neo-Frt on the B6 background. The Frt-neo-Frt sequence was removed using B6;SJL-Tg(ACTFLPe)9205Dym/J transgenic mice purchased from the Jackson laboratory. EnhoIRES-Cre mice were then crossed onto the B6.Cg-Gt(ROSA)26Sortm9(CAG-tdTomato)Hze/J strain in which a loxP-flanked stop cassette prevents transcription of a red fluorescent protein variant (tdTomato) driven by a CAG promoter (5).

### Immunohistochemistry

Surgical anesthesia was achieved using intraperitoneal administration of a cocktail of Ketamine (100 mg/kg) and Xylazine (10 mg/kg), 12 weeks-old *Enho*^*IRES*−*Cre*^*;ROSA-tdTomato* mice were sacrificed by intra-aortic perfusion of 5 ml of ice-cold PBS followed by 50 ml of ice-cold paraformaldehyde 4%. The brain was then removed from the skull, post-fixed overnight in the same fixative, cryoprotected in 30% sucrose solution before being snap freeze in isopentane (−40 °C). Frozen brains were then cut using a cryostat (CM1950, Leica) to collect 30 μm serial sections in PBS.

Immunohistochemistry (IHC) was performed on free-floating sections. To assess Adropin expression in post-mitotic neurons, astrocytes, and endothelial cells antibodies against either NeuN (Abcam cat. # EPR12763; ;1:500 dilution), GFAP (Sigma HPA056030; dilution 1:1000), CD31 (Novus Biologicals NB-80639; 1:100) were used respectively. Briefly, after 1 h in PBS containing 10% Normal Goat Serum (NGS) and 0.2% Triton X100, sections were incubated for 24 h at 4 °C in PBS containing 1% NGS, 0.2% Triton X100 and primary antibody. Goat-Alexa 488 conjugated anti-rabbit IgG (Invitrogen A32731;1:400) was used as secondary antibody. Sections were then mounted on slides and cover slipped using FluorSave reagent (Calbiochem). tdTomato and Alexa 488 fluorescence were visualized with an Olymups confocal using the Fluorview1000 software.

### RNA sequencing and analysis

Total RNA integrity was determined using Agilent Bioanalyzer or 4200 Tapestation. Library preparation was performed with 5–10 ug of total RNA with a Bioanalyzer RIN score >8.0. Ribosomal RNA was removed by poly-A selection using Oligo-dT beads (mRNA Direct kit, Life Technologies). mRNA was then fragmented in reverse transcriptase buffer and heating to 94 degrees for 8 min. mRNA was reverse transcribed to yield cDNA using SuperScript III RT enzyme (Life Technologies, per manufacturer’s instructions) and random hexamers. A second strand reaction was performed to yield ds-cDNA. cDNA was blunt ended, had an A base added to the 3′ ends, and then had Illumina sequencing adapters ligated to the ends. Ligated fragments were then amplified for 12–15 cycles using primers incorporating unique dual index tags. Fragments were sequenced on an Illumina NovaSeq-6000 using paired end reads extending 150 bases.

Samples were prepared according to library kit manufacturer’s protocol, indexed, pooled, and sequenced on an Illumina NovoSeq. Basecalls and demultiplexing were performed with Illumina’s bcl2fastq software and a custom python demultiplexing program with a maximum of one mismatch in the indexing read. RNA-seq reads were then aligned to the Ensembl release 76 primary assembly with STAR version 2.5.1a1. Gene counts were derived from the number of uniquely aligned unambiguous reads by Subread:featureCount version 1.4.6-p52. Isoform expression of known Ensembl transcripts were estimated with Salmon version 0.8.23. Sequencing performance was assessed for the total number of aligned reads, total number of uniquely aligned reads, and features detected. The ribosomal fraction, known junction saturation, and read distribution over known gene models were quantified with RSeQC version 2.6.24.

All gene counts were then imported into the R/Bioconductor package EdgeR5 and TMM normalization size factors were calculated to adjust for samples for differences in library size. Ribosomal genes and genes not expressed in the smallest group size minus one sample greater than one count-per-million were excluded from further analysis. The TMM size factors and the matrix of counts were then imported into the R/Bioconductor package Limma6.

### T-Maze training and testing procedures, novel object recognition

Behavioral experiments were conducted between 0730 and 1400 h. The T-maze is both a learning task based on working-memory and a reference-memory task. The T-maze consisted of a black plastic alley with a start box at one end and two goal boxes at the other. The start box was separated from the alley by a plastic guillotine door that prevented movement down the alley until raised at the onset of training. An electrifiable floor of stainless-steel rods run throughout the maze to deliver a mild scrambled foot-shock.

Mice were not permitted to explore the maze prior to training. A block of training trials began when a mouse was placed into the start box. The guillotine door was raised and a cue buzzer sounded simultaneously; 5 s later, foot-shock was applied. The arm of the maze entered on the first trial was designated “incorrect” and the mild foot-shock was continued until the mouse entered the other goal box, which in all subsequent trials was designated as “correct” for the particular mouse. At the end of each trial, the mouse was returned to its home cage until the next trial.

Mice were trained until they made one avoidance. Training used an inter-trial interval of 60 s, the buzzer was of the door-bell type sounded at 55 dB, and shock was set at 0·35 mA (Coulbourn Instruments scrambled grid floor shocker model E13–08). Retention was tested 1 week later by continuing training until mice reached the criterion of five avoidances in six consecutive trials. The results were reported as the number of trials to criterion for the retention test.

Novel object recognition (NOR) was tested the 5 days following T-maze retention testing. NOR is a declarative memory task that involves the HIP when, as performed here, the retention interval is 24 h after initial exposure to the objects^86^. Mice were habituated to an empty apparatus for 5 min a day for 3 days prior to entry of the objects. During the training session, the mouse was exposed to two identical objects which it was allowed to examine for 5 min. The apparatus and the objects were cleaned between each mouse. 24 h later, the mouse was exposed to one of the original objects and a new novel object in a new location and the amount of time spent examining each object was recorded. The novel object was made from the same material as the original object and of the same size, but a different shape. This eliminated the possibility of smell associated with a particular object being a factor. The underlying concept of the task is based on the tendency of mice to spend more time exploring new, novel objects than familiar objects. The greater the retention/memory at 24 h, the greater the discrimination index (DI). The time with new object (tn) and time spent with the old object (to) was used to calculate the DI [DI = (tn-to)/(tn+to)]^87^.

### Measurements of gene and protein expression

Expression of candidate genes was assessed using qRT-PCR^30^. The extraction of the total RNA from was performed by using kits from Applied Biosystems. cDNA was synthesized with cDNA reverse transcription kit from Quanta. PCR was conducted using a QuantStudio Realtime PCR machine, Applied Systems using primers from Integrated DNA Technology. Data were normalized using three reference genes (HPRT1, 36B4, PPIB). A list of primers and their sequence is provided in Table S1. Quantitative PCR was performed in 384-well plates using SYBR Green and QuantStudio 7 Detection Systems (Applied Biosystems, Life Technologies).

Adropin peptide levels in brain tissues lysate were assessed by Western blot using a mouse monoclonal antibody (Cayman Chemical cat. no. 14117, Ann Arbor, MI; 1:1000 dilution)^40^. An HSP90 rabbit polyclonal antibody (Cell Signaling Technology cat. no. 4874, Danvers, MA; 1:1000 dilution) was used as a reference for loading.

### Statistics

Mouse data were analyzed using Microsoft Excel or Graph Pad Prism software; differences between control and treatment groups were assessed by Student’s *t* test. One-way analysis of variance (ANOVA) and Tukey’s HSD post hoc tests were used to analyze significant differences between control and the three concentrations of adropin for neuritic outgrowth, number of branches, and thickness of primary neurites. Values were considered statistically significant at the level of *p* < 0.05. The data are presented as mean ± S.E.M. Each experiment was replicated a minimum of three times; the actual *N* and *p* values were provided in the text or figure legend.

### Reporting summary

Further information on research design is available in the Nature Research Reporting Summary linked to this article.

## Supplementary information

Reporting Summary

Supplementary Information

## Data Availability

RNA-sequencing data from the experiment investigating the response of 18-month-old C57BL/6J mice to adropin peptide is available from the Gene Expression Omnibus (GSE179088). RNA-sequencing data used to compare ENHO expression in the brain of wild type and transgenic mouse models of dementia were from the Gene Expression Omnibus (GSE125957). Human expression data are available from the Atlas of the Developing Human Brain (www.brainspan.org), Genotype-Tissue Expression (GTEx) portal (https://gtexportal.org/home/), and The Aging, Dementia and TBI Study (http://aging.brain-map.org/). Results from our analysis of the “old-old” dataset downloaded from http://aging.brain-map.org/ are available online (http://pharmacology.slu.edu/results/andrew/TBI_Dementia/). An interactive shiny serve is also available at http://pharmacology.slu.edu/shiny/tbi_shiny/.

## Code availability

No code was generated for this paper.
