# Supplementary material for: Adropin correlates with aging-related neuropathology in humans and improves cognitive function in aging mice
Source: NPJ Aging Mech Dis. 2021 Aug 30;7:23. doi: 10.1038/s41514-021-00076-5 (PMC8405681; doi:10.1038/s41514-021-00076-5)
Supplement: Supplementary file 1 — Reporting Summary [file 41514_2021_76_MOESM1_ESM.pdf]

nature research

Corresponding author(s): Andrew Butler
Susan Farr

Last updated by author(s): Jul 1, 2021

Reporting Summary

Nature Research wishes to improve the reproducibility of the work that we publish. This form provides structure for consistency and transparency in reporting. For further information on Nature Research policies, see our Editorial Policies and the Editorial Policy Checklist.

Statistics

For all statistical analyses, confirm that the following items are present in the figure legend, table legend, main text, or Methods section.

n/a	Confirmed	
☐	☒	The exact sample size (*n*) for each experimental group/condition, given as a discrete number and unit of measurement
☐	☒	A statement on whether measurements were taken from distinct samples or whether the same sample was measured repeatedly
☐	☒	The statistical test(s) used AND whether they are one- or two-sided<br>*Only common tests should be described solely by name; describe more complex techniques in the Methods section.*
☐	☒	A description of all covariates tested
☒	☐	A description of any assumptions or corrections, such as tests of normality and adjustment for multiple comparisons
☐	☒	A full description of the statistical parameters including central tendency (e.g. means) or other basic estimates (e.g. regression coefficient) AND variation (e.g. standard deviation) or associated estimates of uncertainty (e.g. confidence intervals)
☒	☐	For null hypothesis testing, the test statistic (e.g. *F*, *t*, *r*) with confidence intervals, effect sizes, degrees of freedom and *P* value noted<br>*Give P values as exact values whenever suitable.*
☒	☐	For Bayesian analysis, information on the choice of priors and Markov chain Monte Carlo settings
☒	☐	For hierarchical and complex designs, identification of the appropriate level for tests and full reporting of outcomes
☐	☒	Estimates of effect sizes (e.g. Cohen's *d*, Pearson's *r*), indicating how they were calculated

Our web collection on statistics for biologists contains articles on many of the points above.

Software and code

Policy information about availability of computer code

Data collection	No software was used
Data analysis	No code was used

For manuscripts utilizing custom algorithms or software that are central to the research but not yet described in published literature, software must be made available to editors and reviewers. We strongly encourage code deposition in a community repository (e.g. GitHub). See the Nature Research guidelines for submitting code & software for further information.

Data

Policy information about availability of data

All manuscripts must include a data availability statement. This statement should provide the following information, where applicable:
- Accession codes, unique identifiers, or web links for publicly available datasets
- A list of figures that have associated raw data
- A description of any restrictions on data availability

All data are available on request. RNA seq data will be deposited to the GEO prior to publication.

Field-specific reporting

Life sciences study design

All studies must disclose on these points even when the disclosure is negative.

Sample size	For the mouse experiments, we used an n=15 per group for the adropin peptide treatment study. N's of 10-13 were used for the adropin transgenic studies.
Data exclusions	No data was excluded.
Replication	replication involved performing animal studies using two different models (transgenic, peptide treatment). Cell culture studies were performed twice
Randomization	Transgenic mice assigned themselves to groups ("wild-type" littermate controls and mice positive for the transgene). 18-month-old mice purchased from the Jackson Laboratory were weighed prior to being assigned into two groups matched for body weight.
Blinding	Dr. Sue Farr was blinded during the mouse behavioral experiments (measurement of learning and memory, novel object recognition)

Reporting for specific materials, systems and methods

We require information from authors about some types of materials, experimental systems and methods used in many studies. Here, indicate whether each material, system or method listed is relevant to your study. If you are not sure if a list item applies to your research, read the appropriate section before selecting a response.

Materials & experimental systems

n/a	Involved in the study	
☐	☒	Antibodies
☐	☒	Eukaryotic cell lines
☒	☐	Palaeontology and archaeology
☐	☒	Animals and other organisms
☒	☐	Human research participants
☐	☒	Clinical data
☒	☐	Dual use research of concern

Methods

n/a	Involved in the study	
☒	☐	ChIP-seq
☒	☐	Flow cytometry
☒	☐	MRI-based neuroimaging

Antibodies

Antibodies used	Cayman Chemical cat. no. 14117 (adropin); Cell Signaling Technology cat. no. 4874 (HSP90); CAT#: NB600-1475 (CD31); EPR12763 (NeuN); ZRB2383 (GFAP)
Validation	14117: tested using recombinant protein, over expression vector and in knockout models (DOI:https://doi.org/10.1074/jbc.RA120.014381).<br>4874: see https://www.cellsignal.com/products/primary-antibodies/hsp90-antibody/4874<br>NB600-1475 (CD31): see https://www.novusbio.com/products/cd31-pecam-1-antibody-mec133_nb600-1475<br>EPR12763: see https://www.abcam.com/neun-antibody-epr12763-neuronal-marker-ab177487.html<br>ZRB2383: seehttps://www.sigmaaldrich.com/catalog/product/sigma/zrb2383?

Eukaryotic cell lines

Policy information about cell lines

Cell line source(s)	Primary cultured mouse hippocampal neurons
Authentication	None needed - they are primary culture
Mycoplasma contamination	Not tested
Commonly misidentified lines (See ICLAC register)	None were used

Animals and other organisms

Policy information about studies involving animals; ARRIVE guidelines recommended for reporting animal research

Laboratory animals	C57BL/6J mice
Wild animals	N/A

Field-collected samples	N/A
Ethics oversight	Saint Louis University IACUC

Note that full information on the approval of the study protocol must also be provided in the manuscript.

Clinical data

Policy information about clinical studies

All manuscripts should comply with the ICMJE guidelines for publication of clinical research and a completed CONSORT checklist must be included with all submissions.

Clinical trial registration	N/A
Study protocol	N/A
Data collection	N/A
Outcomes	N/A
