# Supplementary material for: Adropin correlates with aging-related neuropathology in humans and improves cognitive function in aging mice
Source: NPJ Aging Mech Dis. 2021 Aug 30;7:23. doi: 10.1038/s41514-021-00076-5 (PMC8405681; doi:10.1038/s41514-021-00076-5)
Supplement: Supplementary file 2 — Supplementary Information [file 41514_2021_76_MOESM2_ESM.pdf]

Table S1. Primer sequences for qRT-PCR.

QPCR primers mouse	
**Sequence (5'->3')**	
AGCTGTAGTTTTTGTCACCA	m CCL2 FP
TGTCTGGACCCATTCCTTCT	m CCL2 RP
GGAGAGCTTCATCTGTGTCTC	m IL17 FP
GCTGAGCTTTGAGGGATGAT	m IL17 RP
GCCAAGATCTGTGTCTCTCC	m IL13 FP
AGGGGAGTCTGGTCTTGTG	m IL13 RP
TCACTGTAGATGGGCAATGG	m MMP28 FP
CTCAATACCAGGTGGCAGTC	m MMP28 RP
GCGACAGGTGTCCTTGGAAT	m FGFR3 FP
CCAGAACAGGACCTTCTCCT	m FGFR3 RP
GAAGCGAAGGACGGATCTTG	m CTSL FP
AACCCCATGGTCGAGGTTCT	m CTSL RP
TGCCACCTTTTGACAGTGAT	m IL1β FP
GAAGCAGCCCTTCATCTTTTG	m IL1β RP
AGCAGTCTTGACGCAGACCT	m STK11 FP
CAAAGTCACCAAGTGCTCCA	m STK11 RP
CTCAGTGAAGAACAGGCACAA	m VLCAD FP
CTTGGCAGGGTCATTCACTT	m VLCAD RP
CAAGAAGTGTCTACAGCCTGC	m SNTA1 FP
CTGAAGTTTCTCGAAGGGCT	m SNTA1 RP
TCCGGCCCGAGATGAAT	m NAMPT FP
GTGGGTATTGTTTATAGTGAGTAACCTTGT	m NAMPT RP
TGACTTTGCCGAGAAGGAGT	m ACADS FP
ACTCAGCTCCTCTGGCACAT	m ACADS RP
GGAGCTCCAAGACTCTAGACA	m PPARGC1α FP
CCAAAGTCTCTCTCAGGTAGC	m PPARGC1α RP
GGTGTTTGGCGCAGCACCTT	m NRF1 FP
CTCTGGGATAAATGCCCGAAGCT	m NRF1 RP
CGGTGAGCCTGGCCT	m CPT1a FP
CATCTTGAGTGGTGACCGAG	m CPT1a RP
AGTGTGCAGACAGACCATTG	m TNS3 FP
GACTCTCAGGAGCCTGGTC	m TNS3 RP
ATCTTTTCCTCGGAGCATGA	m LCAD FP
TTTCTCTGCGATGTTGATGC	m LCAD RP
CAGGGAACTTTGCGAGTGA	m S1PR1 FP
AAACAGCAGCCTCGCTCAA	m S1PR1 RP
ACAGGGGGTTCCAGCTGACCA	m PARK2 FP
TCCACCGGCAGGGTATGGCT	m PARK2 RP
GAGCCGAGAGTGGGGCTTTGC	m ULK1 FP
GCCCTGGCAGGATACCACGC	m ULK1 RP
CTTCTAAGTCACCCACACCTG	m GPAM FP
CTTACTGGTCCTGTATCCTTGA	m GPAM RP
GCAAAGGATGATTCGGCTCAGGGAA	m TFAM FP
CCGGATCGTTTCACACTTCGACGG	m TFAM RP
ACCAGGTCATCAGTACACCA	m PRKAA1 FP
CCTTTTCGTCCAACCTTCCA	m PRKAA1 RP
GCAACAATTCCTGGCGTTAC	m TGFβ FP
CCTGTATTCCGTCTCCTTGG	m TGFβ RP
ACTATACTACTAACAGACCG	m mtCO1 FP
GGTTCTTTTTTTCCGGAGTA	m mtCO1 RP
GTTGGTCCATACGGCATTTT	m mtND1 FP
TGGGTGTGGTATTGGTAGGG	m mtND1 RP
TCTCCTGTGGGATTCCTGAC	m SIRT1 FP
ACACAGAGACGGCTGGAACT	m SIRT1 RP
TTTGCAACTCAAGTCCTGGT	m CPT1c FP
ACGTACAGAAAATCCATCATGTAG	m CPT1c RP
AGCAGCTCTGGATGGGACTGC	m ATG5 FP
GCCGCTCCGTCGTGGTCTGA	m ATG5 RP
AACCATAGGGCACCAATGATAC	m mtCO2 FP
GGATGGCATCAGTTTTAAGTCC	m mtCO2 RP
GCTCCTGGGTAGAACTGCAC	m BNIP3 FP
GCTGGGCATCCAACAGTATT	m BNIP3 RP
TCCCCAAAGGGATGAGAAGT	m TNFα FP
CCACTTGGTGGTTTGTGAGT	m TNFα RP
CATCAACAGAGGGTGCCAAC	m SLC1A2 FP
CAGTCAGTGAGAGCAGGAGA	m SLC1A2 RP
GCCTCCGAAACCATGAACTT	m VEGF FP
ACTTGATCACTTCATGGGACT	m VEGF RP
AGGGCGCGAACCCCTG	m CNTFR FP
CAGCTGTTCTCCACACACAT	m CNTFR RP
AGCTCCGGGTTGGTATACTG	m BDNF FP
TGGTGGAACTTCTTTGCGG	m BDNF RP
ATATGAAGTGACCAACCGCC	m eNOS FP
GGAGCTGAAAACCTCATCTGT	m eNOS RP
AGTGTCTGGAGTTAAGGCCC	m PPP1R1B FP
CCTCTCCAGAGGTTCTCTGAT	m PPP1R1B RP
ACCGCCTGGTTCTCCTATAA	m 36B4 FP
AAGACGATGTCACTCCAACG	m 36B4 RP
CAGTACAGCCCCAAAATGGT	m HPRT FP
AATCCAACAAAGTCTGGCCT	m HPRT RP
CTCTCGGAGCGCAATATGAA	m PPIB FP
AAGTATACCTTGACTGTGACTTT	m PPIB RP

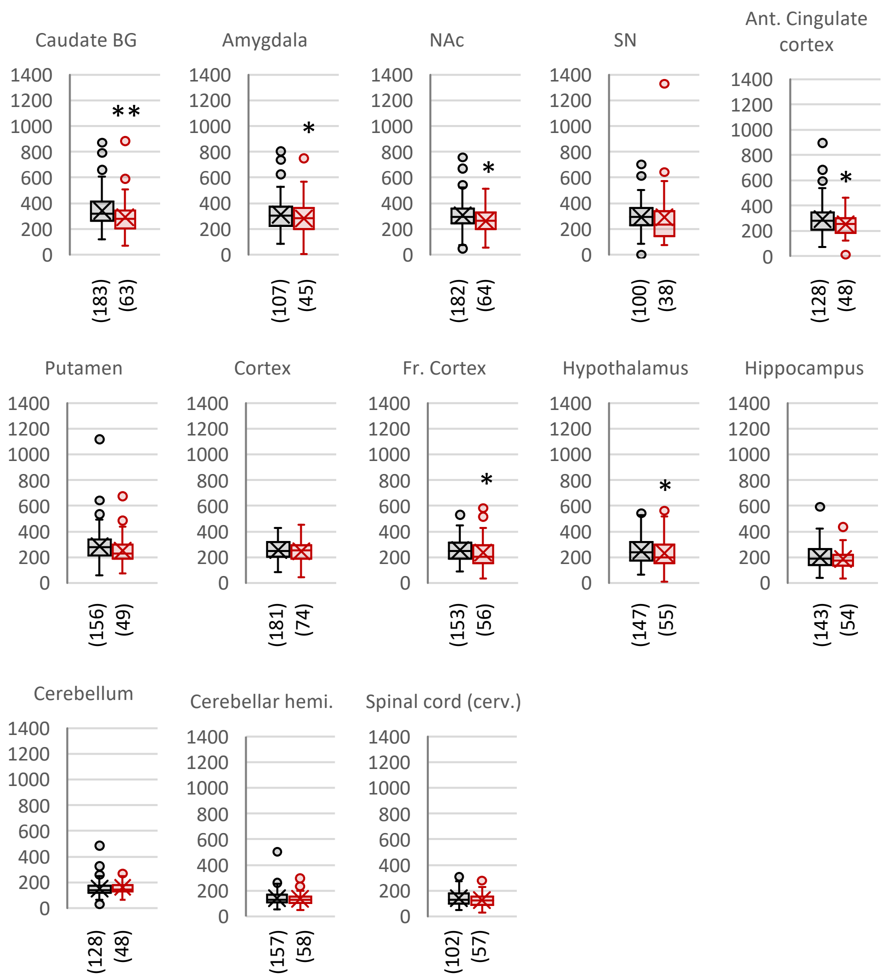

Figure S1. Comparisons of *ENHO* expression (in TPM) in males (grey) and females (red).

Data (TPM) were downloaded from the GTEx portal. are Sample size are shown in brackets. ** p<0.01, * p<0.05

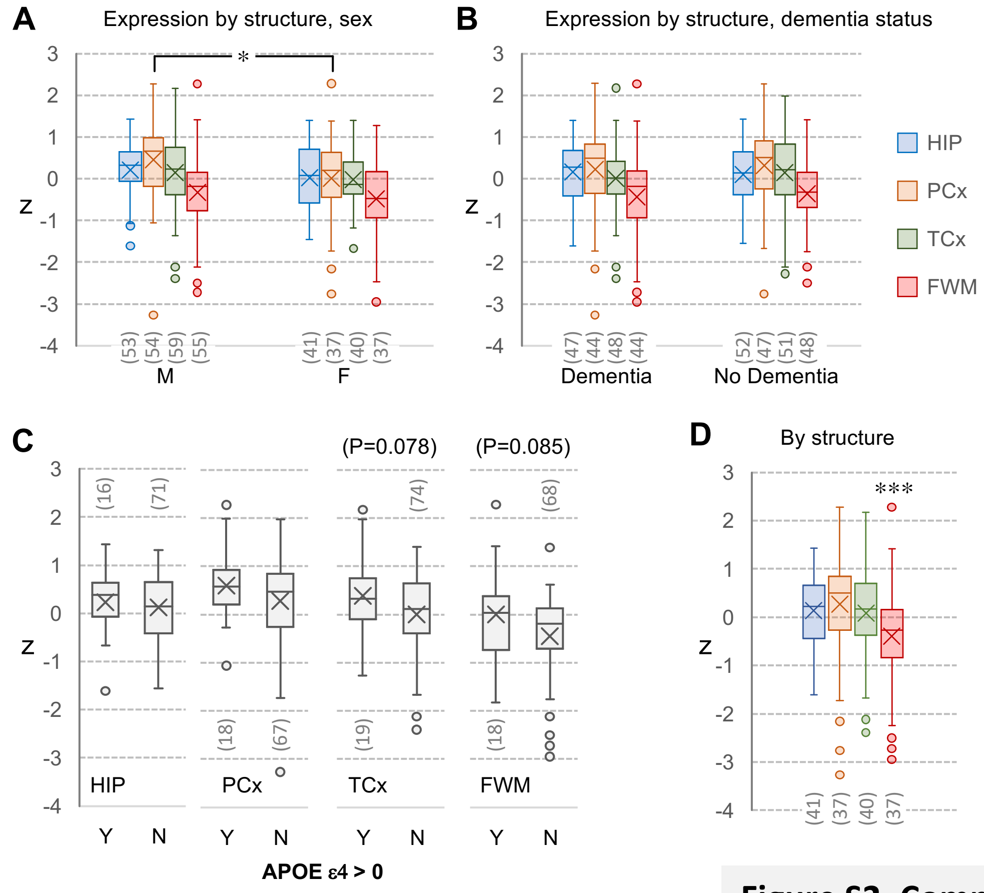

E

	Correlation coefficients (r)					
	DEMENTIA			NO DEMENTIA		
	PCx	TCx	FWM	PCx	TCx	FWM
**HIP**	0.555	0.385	0.483	0.559	0.576	0.169
**PCx**		0.655	0.774		0.700	0.317
**TCx**			0.627			0.222

Figure S2. Comparison of *ENHO* expression in the aged human brain. Data shown compared expression beween structures by sex (A), dementia status (B), grouped by APOE ε4 varian (C), between structures (D). (E-K) correlations between structures. *p<0.05 between groups shown; *** p<0.001 vs. PCx. Sample size are shown in brackets.

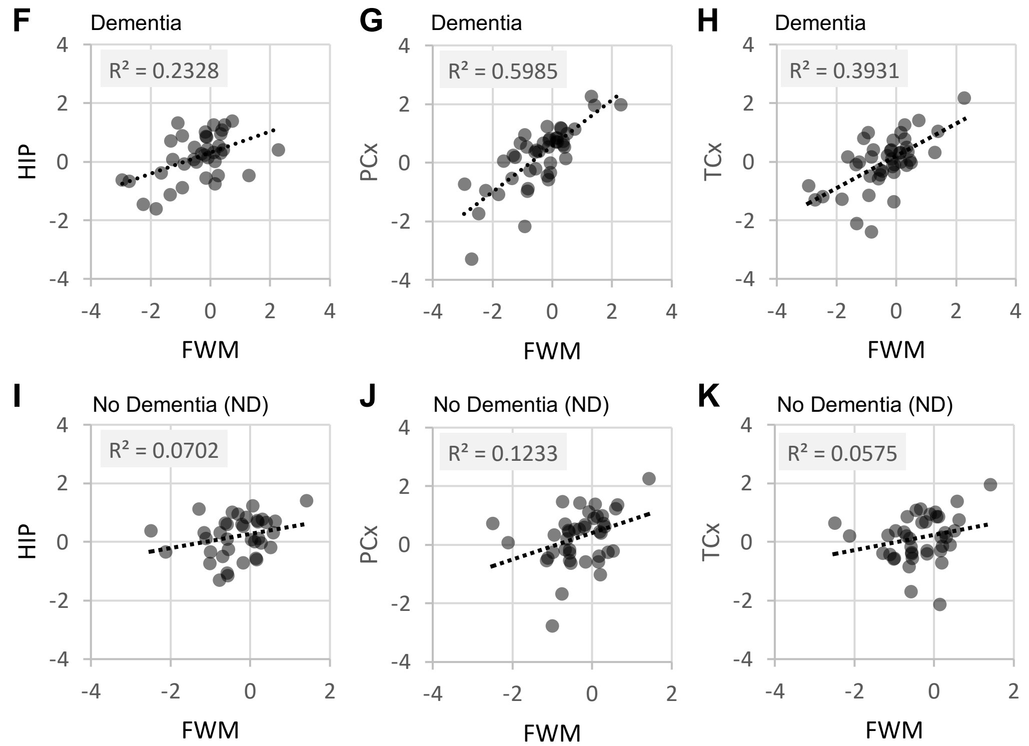

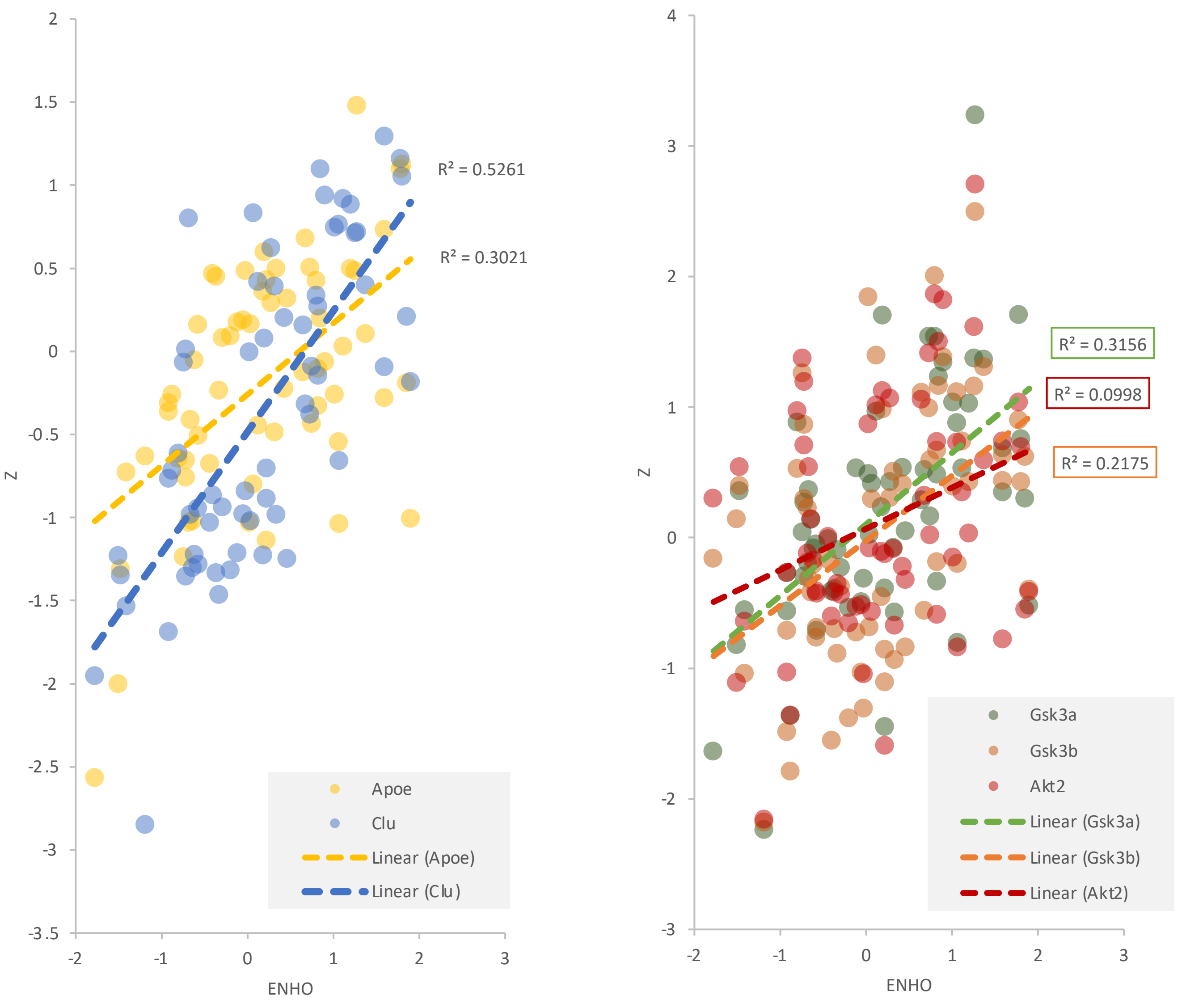

Figure S3. Relationships between the expression of ENHO and genes showing high expression in astrocytes (Apoe, Clu) or insulin signaling (Gsk3a/b, Akt2) in the brains of wild type mice (n=63). The data shown are z scores derived from expression data downloaded from GSE125957 (Castanho et al. Transcriptional Signatures of Tau and Amyloid Neuropathology *Cell Rep*. 2020 Feb 11; 30(6): 2040–2054.e5). RNA seq used total RNA from the entorhinal cortex (left hemisphere) from mice aged 2-12 months. The animal procedures were carried out at Eli Lilly and Company in accordance with the UK Animals (Scientific Procedures) Act of 1986 and where approved by the local Animal Welfare and Ethical Review Board. These data are from female mice on mixed backgrounds (C57BL/6JOlaHsd; 129S6/SvEvTac + FVB/NCrl).

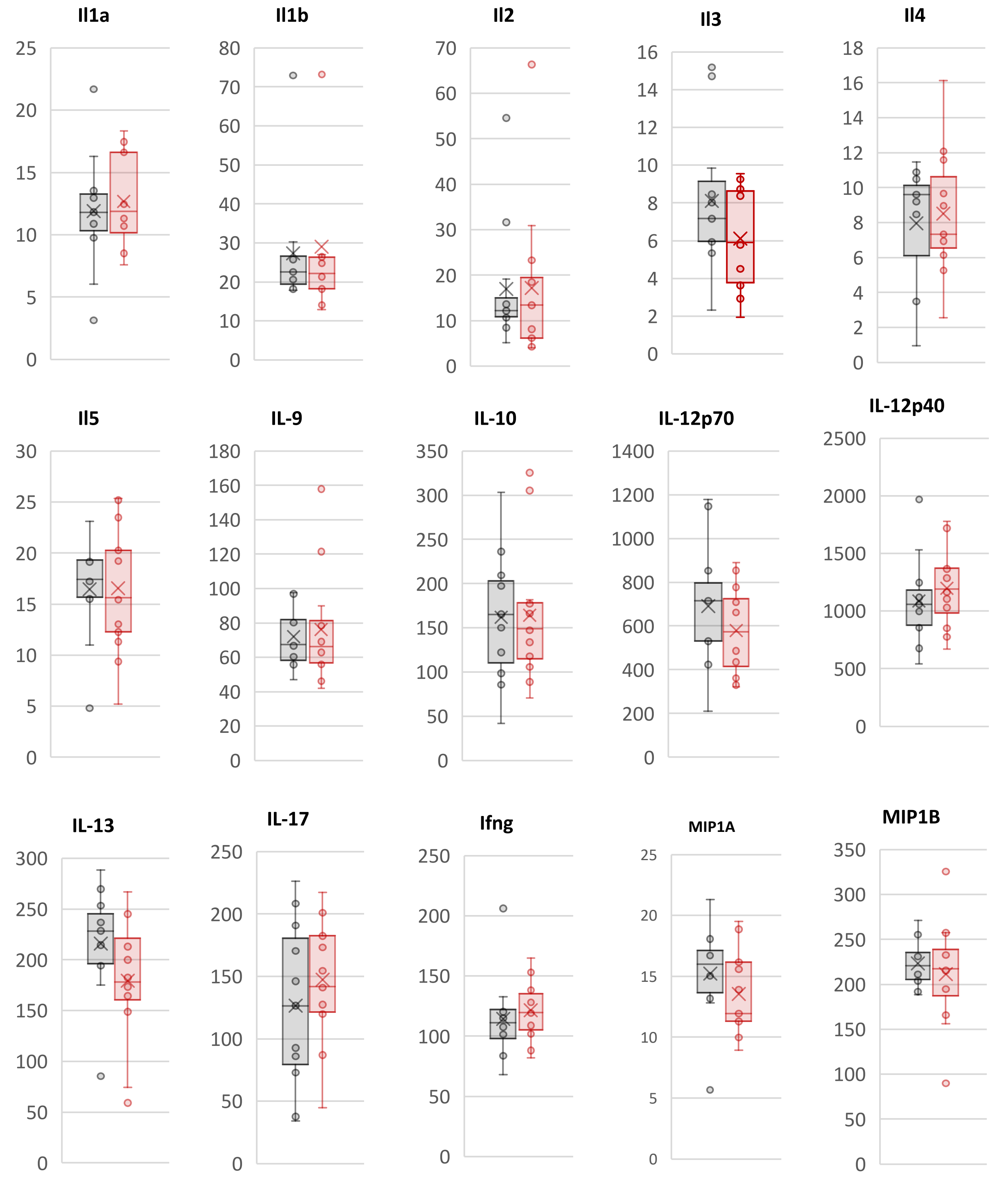

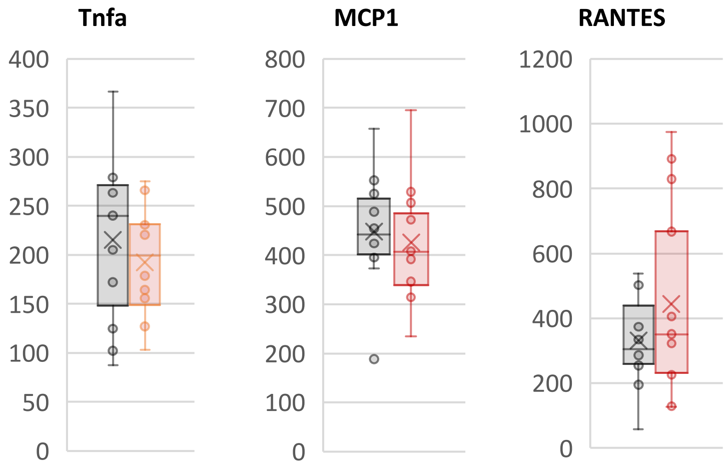

Figure S4. Serum cytokine concentrations in male 18-month-old WT (grey, n=13) and AdrTG (red, n=11) mice.

A

		control	tmt
start weight (g)	mean	43.7	43.0
	median	41.8	42.0
	SD	6.9	7.0
end weight (g)	mean	44.2	44.2
	median	40.6	42.1
	SD	8.0	8.4
delta (g)	mean	0.5	0.7
	median	0.3	-0.2
	SD	2.5	3.2
Blood glucose mg/dL	mean	203	194
	median	203	184
	SD	22	31
Insulin ng/ml	mean	2.7	4.8
	median	1.7	2.2
	SD	1.9	4.8

B

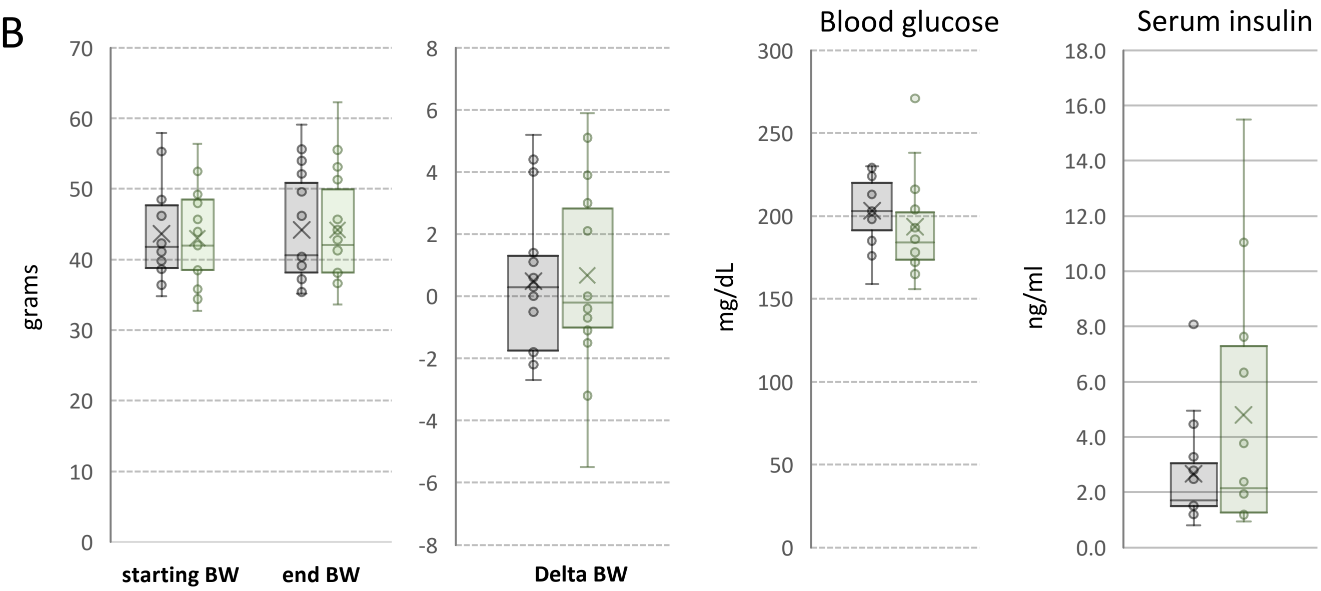

Figure S5. Body weight (A, B) and blood chemistry (C) data of 18-month-old mice treated with 0.9% saline (controls, grey) or 90 nmol/kg/d adropin$^{34\text{-}76}$ (green) for 4 weeks (n=15/group).

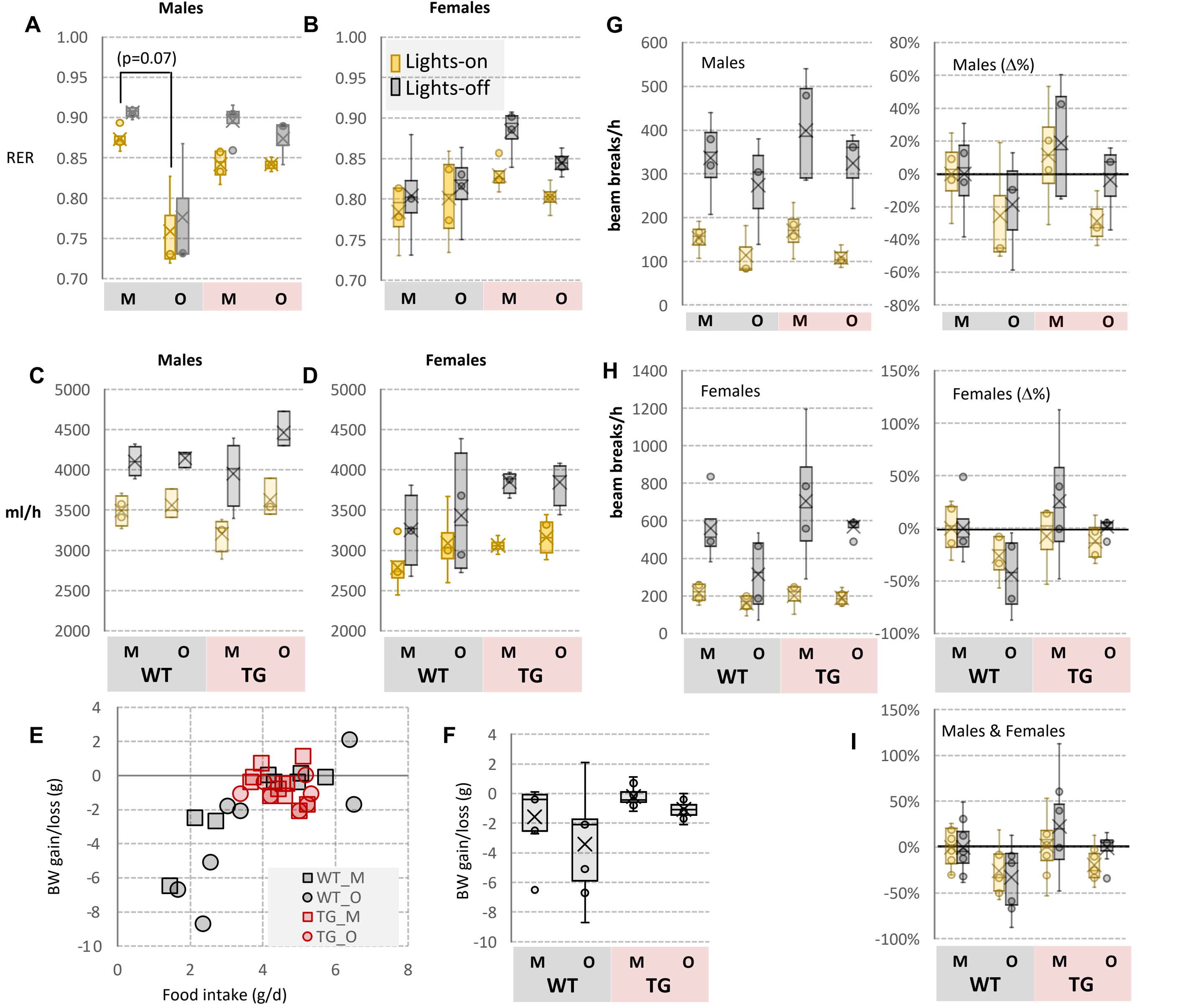

Figure S6. Assessment of whole-body energy balance using indirect calorimetry, food intake and ambulatory activity in 'middle-aged' ('M', 8-10 months) or 'old' ('O', 16-18 months) AdrTG and WT controls.

Mice of the age and genotype indicated were housed in metabolic chambers for 3d to measure fuel selection (respiratory exchange ratio, RER), energy expenditure (VO_2) and physical activity. Food intake was estimated by measuring food weights at the start and end of the study.

(A, B) Fuel selection indicated by RER. Old male WT mice have lower RER, suggesting fat oxidation. (C, D) Oxygen consumption in ml/h. (E) Weight change during the recording as a function of food intake group by age and genotype. Mice eating less food lost weight during the recording period. (F) Weight change in mice grouped by age and genotype – old WT mice lost more weight during the recording period. (G-H) Ambulatory activity is shown as actual values (left panel) or % of M for WT and AdrTG (right panel). WT mice of either sex show a decline in ambulatory activity (25%) that is not observed in AdrTG. (I) Ambulatory activity data pooled from male and female mice.

WT=wild type; AdtTG=adropin transgenic. Sex is shown at the top of the chart.

WT-M, n=4; WT-O, n=3 (males) or 4 (females); AdrTG-M, n=4; AdrTG-O, n=3 (males) or 4 (females).

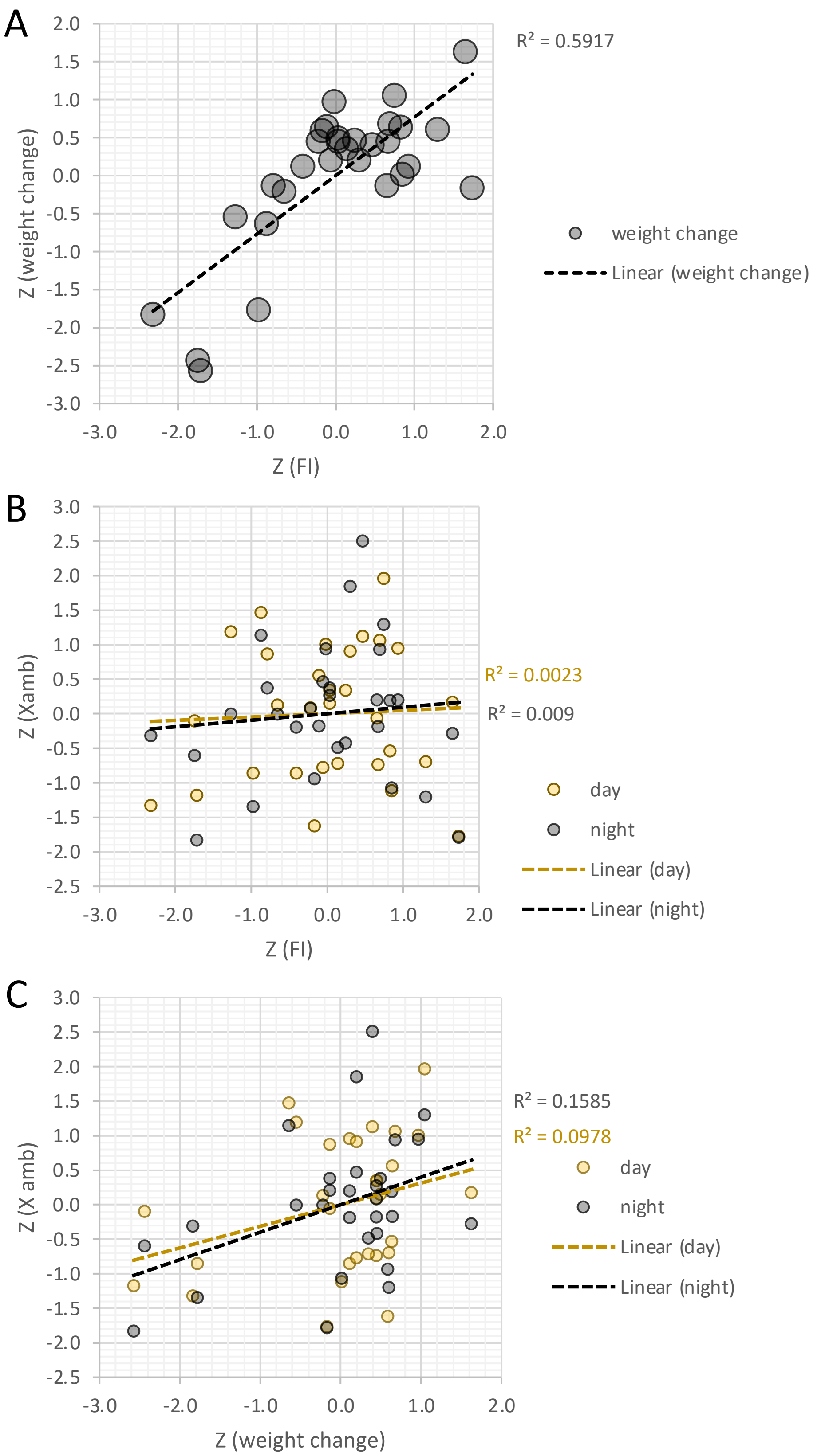

Figure S7. Variations in food intake predicts weight gain/loss, however variation in weight or gain loss is a a weak predictor of X ambulatory activity.

The data are expressed as Z scores calculated for male and female mice. Food intake is a strong predictor changes in body weight during the recording phase (A). However, there is no correlation between food intake and X ambulatory activity (B), and at best a weak correlation between energy balance (indicated by weight gain or loss) and X ambulatory activity (C).

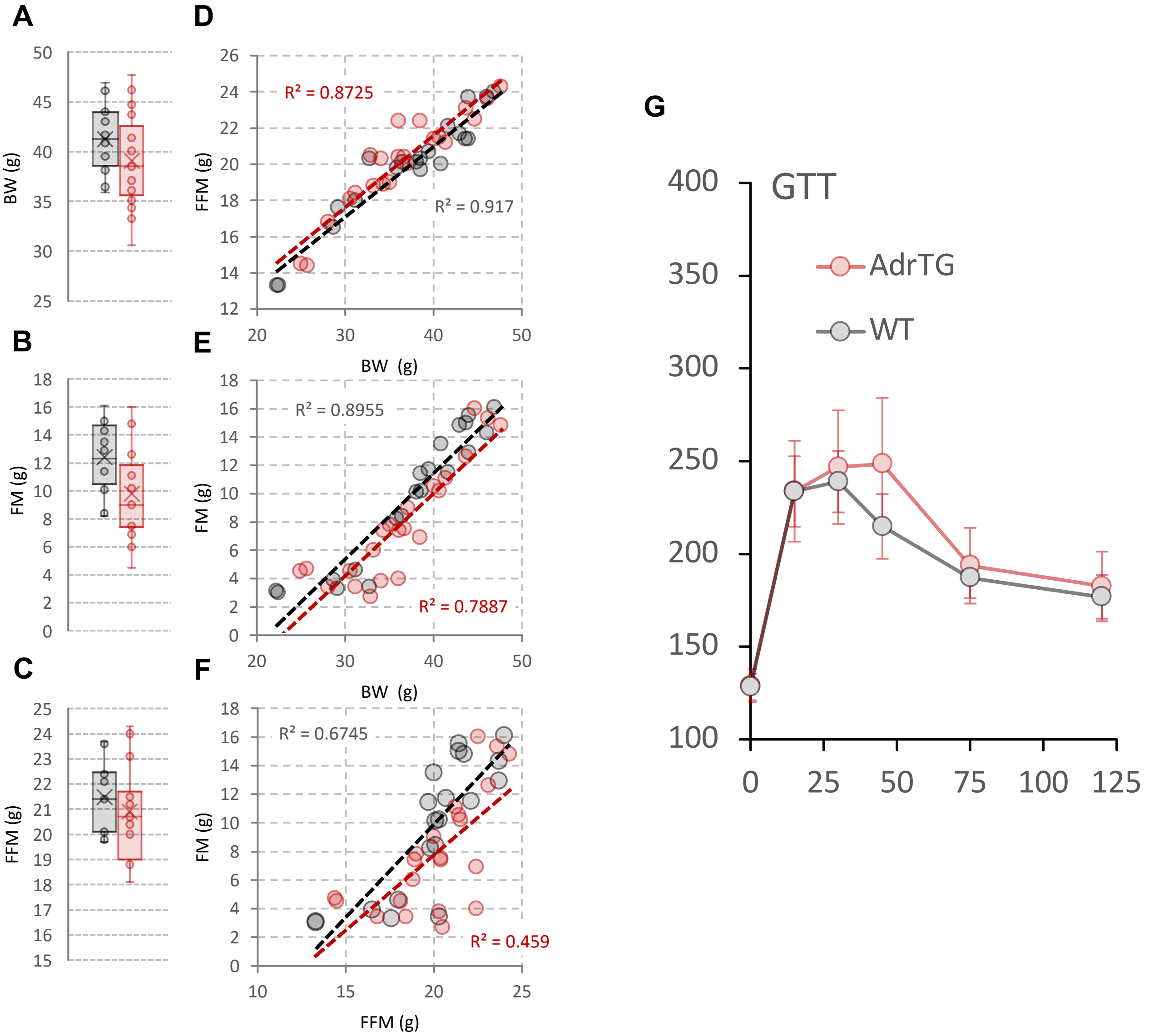

Figure S8. Body weight (A) and composition (B-F) and glucose tolerance test results (G) for 18-month-old male AdrTG (red) and WT (black) controls. Measurements of body weight and composition (fat free mass (FFM); fat mas (FM)) were unremarkable in AdrTG. Using a regression approach suggests subtle differences between genotype, with a lower FM expressed as a function of BW (F) or FFM (F). Glucose tolerance was not affected by genotype.
